# Supplementary material for: LETM1 couples mitochondrial DNA metabolism and nutrient preference
Source: EMBO Mol Med. 2018 Jul 16;10(9):e8550. doi: 10.15252/emmm.201708550 (PMC6127893; doi:10.15252/emmm.201708550)
Supplement: Supplementary file 1 — Appendix [file EMMM-10-e8550-s001.pdf]

APPENDIX

Table of Contents

1) Legends to Appendix Figures
2) Appendix Figure S1
3) Appendix Figure S2
4) Appendix Figure S3
5) Appendix Figure S4
6) Appendix Figure S5
7) Appendix Figure S6
8) Appendix Figure S7
9) Appendix Figure S8
10) Appendix Figure S9
11) Appendix Table S1
12) Appendix Table S2
13) Appendix Table S3
14) Appendix Table S4
14) Appendix Table S5

Legends to Appendix Figures

Appendix Figure S1. Mitochondrial translation and transcript levels after siRNA mediated silencing of human *LETM1*. (A) Schematic representation of the human *LETM1* gene showing the locations of the siRNA oligos used to target *LETM1* in this study. **(B)** 35S labeling of *de novo* mitochondrial protein synthesis in HeLa cells 72 h (1 round) or 144 h (2 rounds) after transfection with a non-target dsRNA (NT), or a siRNA targeting the assembly factor of the 55S ribosome *MPV17L2* (L2), or *LETM1* (siR1 or siR2) for 144 h. Immunoblots for LETM1 indicate the efficiency of KD and coomassiestained gels are shown as loading controls. **(C)** Immunoblot analysis of mitochondrial ribosomal subunit MRPL11 in HeLa cells treated with siNT or *LETM1* siR1, siR2 or siR3 144 h. (**D)** The abundance of the 5 individual mitochondrial mRNAs (y axis) relative to mtDNA copy number (x axis) whose mean value appears in Fig 2E.

Appendix Figure S2. Mitochondrial DNA after *LETM1* silencing in HeLa cells. Immunofluorescence analysis of HeLa cells treated with siR2 showing DNA (green), and, additionally, in the merged images TOM20 (red) and nuclear DNA (blue). Scale bars 12 μm, and in the higher magnifications below, 4 μm.

Appendix Figure S3. Changes in DRP1 activity in response to *LETM1* silencing. (**A)** Steady state levels of LETM1, DRP1S616, and total DRP1 in whole HeLa cell extracts 72 h after transfection with either NT, or with siR1 or siR2, with GADPH as loading control. (**B)** 35S labeling of *de novo* mitochondrial protein synthesis for 1 h in HeLa cells treated with siNT, siR1 (targeting *LETM1*), siD (targeting *DRP1*) or both siR1 and siD, 144 h after the first of two rounds of transfection. A coomassie stained gel is shown as a loading control.

Appendix Figure S4. Chromosome mapping of 4p deletions in the Wolf-Hirschhorn syndrome critical region-2 (WHSCR-2) of subjects S1-S5. To note, the deletions in patients S1-S4 include *LETM1* (in red*),* which is spared in subject S5.

Appendix Figure S5. Mitochondrial protein synthesis and OXPHOS subunit levels in WHS fibroblasts. (A) 35S labeling of *de novo* mitochondrial translation in controls (C1-C4), WHS *LETM1+/-* (S1-S4) or WHS *LETM1+/+* S5 fibroblast cells. Coomassie stained proteins are shown as loading controls. **(B)** Immunoblots of OXPHOS proteins in whole cell extracts from control (C1-C3), WHS *LETM1+/-* (S1-S4) and WHS *LETM1+/+* S5 fibroblasts, with GAPDH as loading control.

Appendix Figure S6. Mitochondrial morphology and DNA distribution in WHS *LETM1+/-* fibroblasts. (A) Immunofluorescence analysis of WHS *LETM1+/-* S1, S3 fibroblasts labeled with anti-TOM20 antibody. **(B)** Confocal images of WHS *LETM1+/-* S1 and S3 cells labeled with anti-TOM20 (red) and anti-DNA (green), and DAPI (blue). Scale bars 15 µm and 7 µm in the higher magnifications.

Appendix Figure S7. Immunodetection of the inactive (phosphorylated) form of PDH, PDHS293, in HeLa cells after *LETM1* silencing with siR1. LETM1 antibody indicates the extent of repression and GAPDH is shown as loading control. NT – nontarget control siRNA.

Appendix Figure S8. Growth of WHS *LETM1+/-* fibroblasts in ketone body supplemented medium. (**A**) Light microscope images of S2 derived fibroblasts growing in 0.3 mM BHB with and without passaging. The cells continue to grow up to day 21, when the experiment was terminated.

Appendix Figure S9. Mitochondrial DNA in S2 fibroblasts grown on ketone bodies in place of glucose and pyruvate. Immunofluorescence analysis of WHS *LETM1+/-* S2 fibroblasts labeled with anti-DNA (green), and anti-DNA and TOM20 (merge), after 2 days (A) and 21 days (B) growth on 0.3 mM BHB. Scale bars 15 µm.

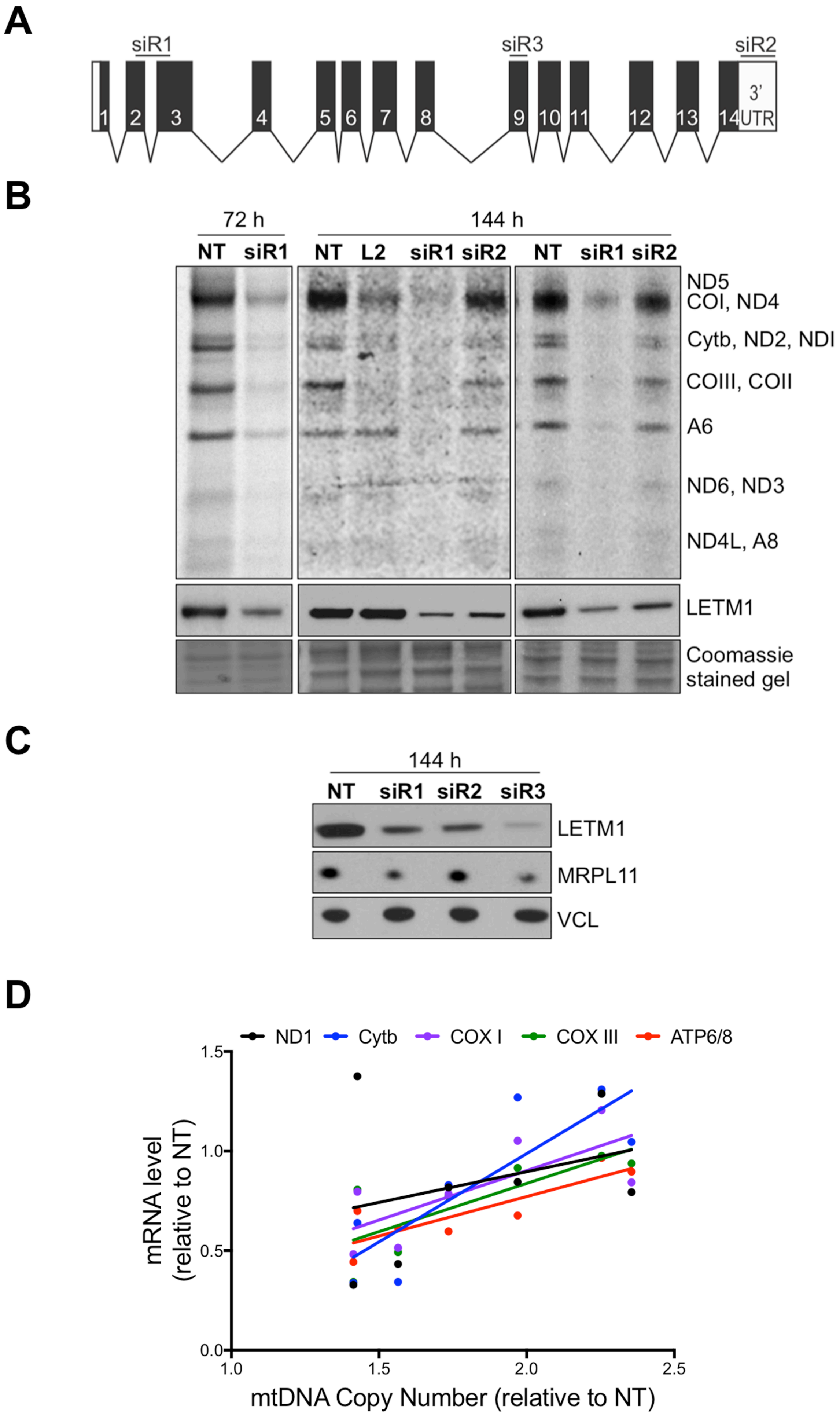

Appendix Figure S1

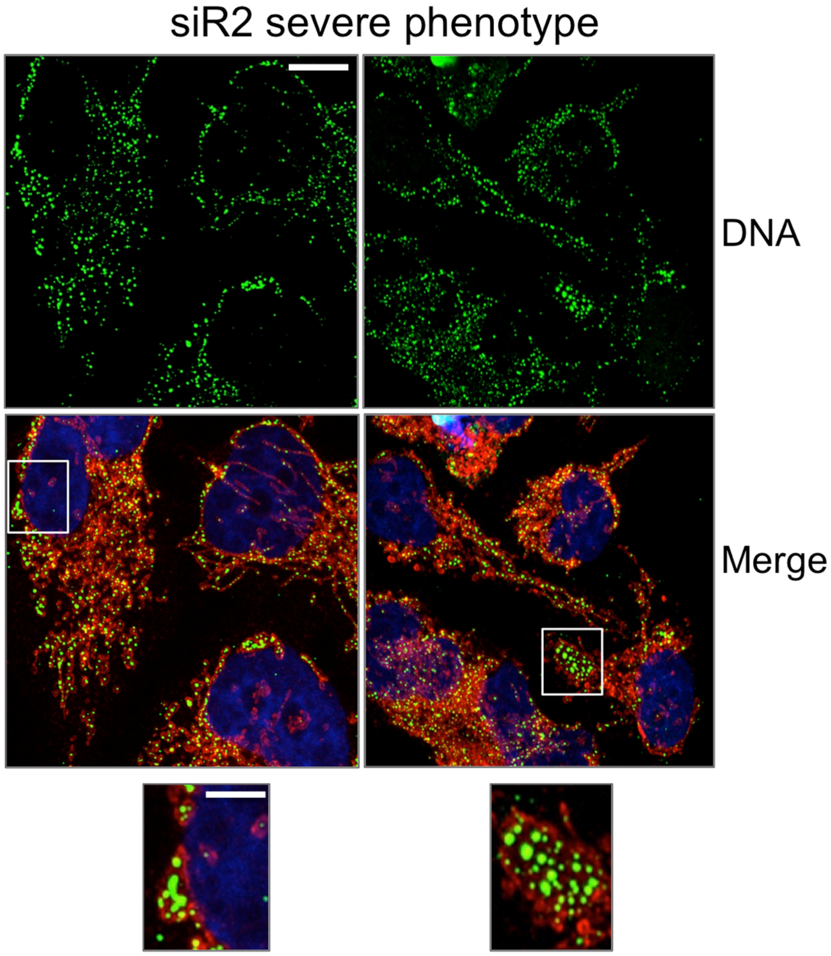

Appendix Figure S2

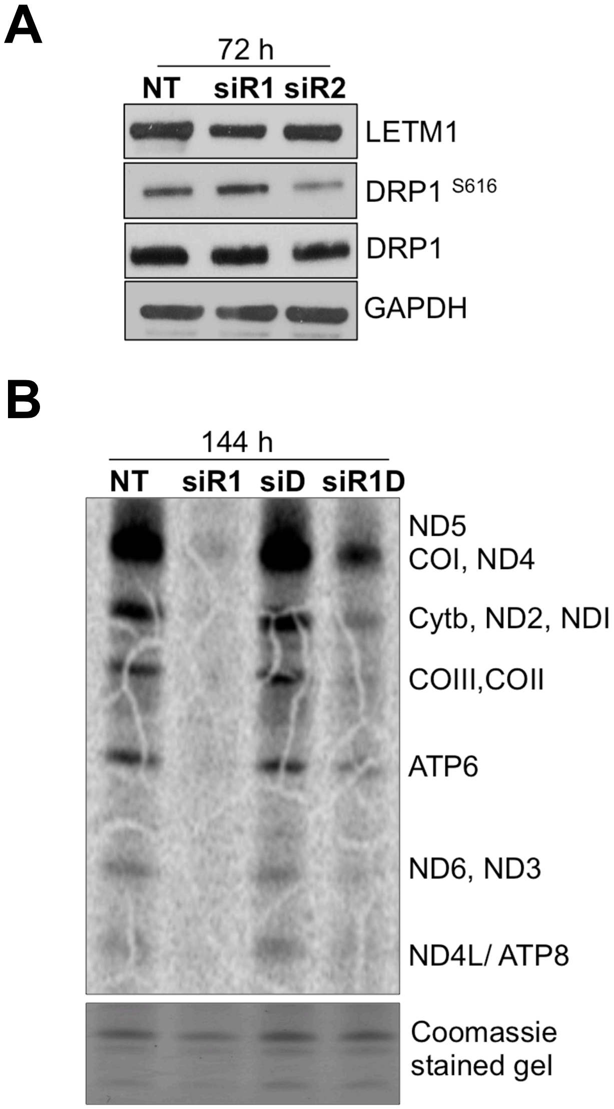

Appendix Figure S3

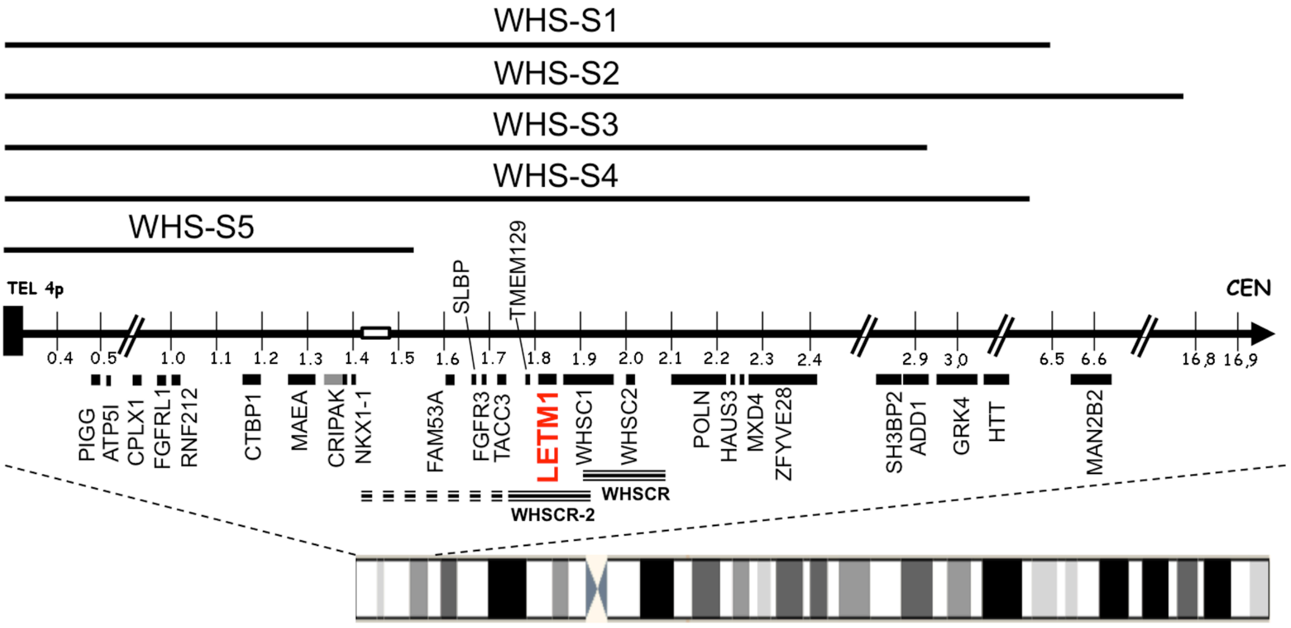

Appendix Figure S4

A

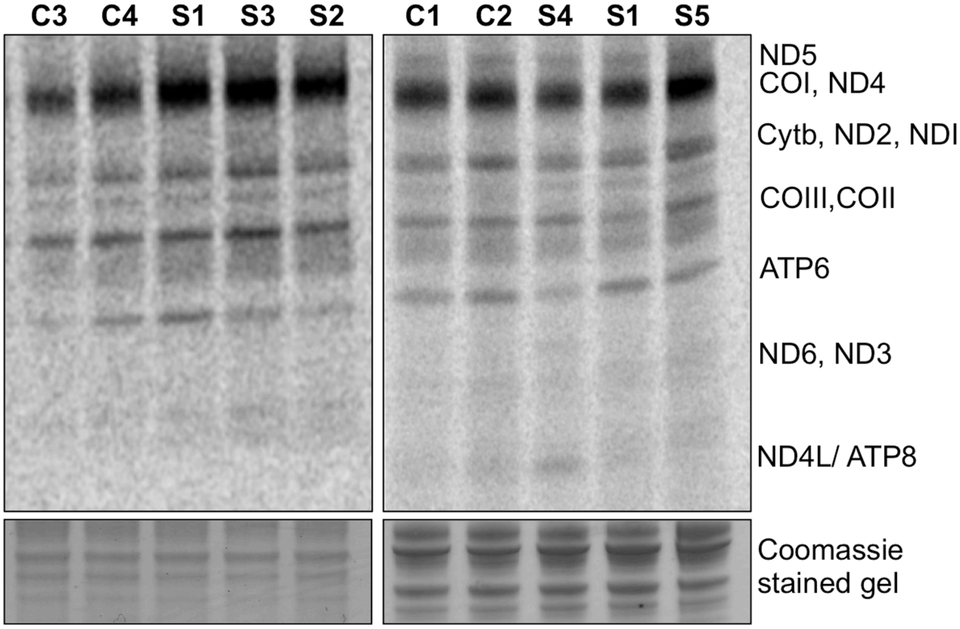

B

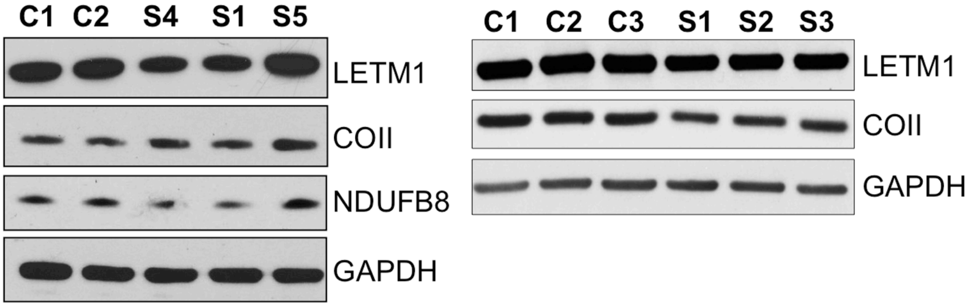

Appendix Figure S5

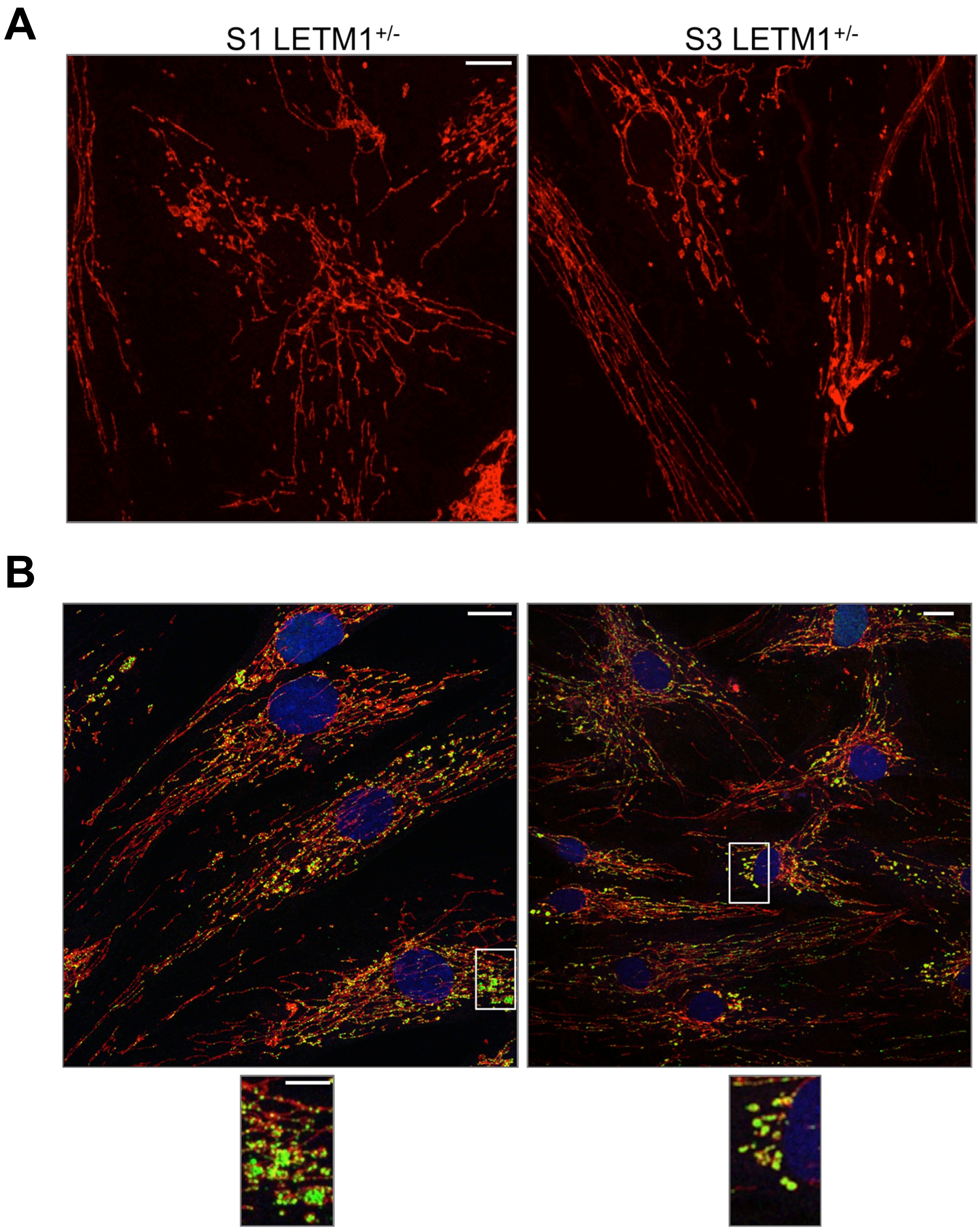

Appendix Figure S6

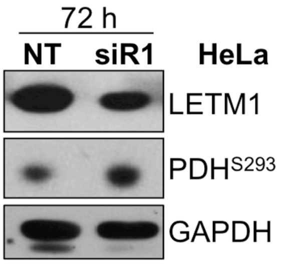

Appendix Figure S7

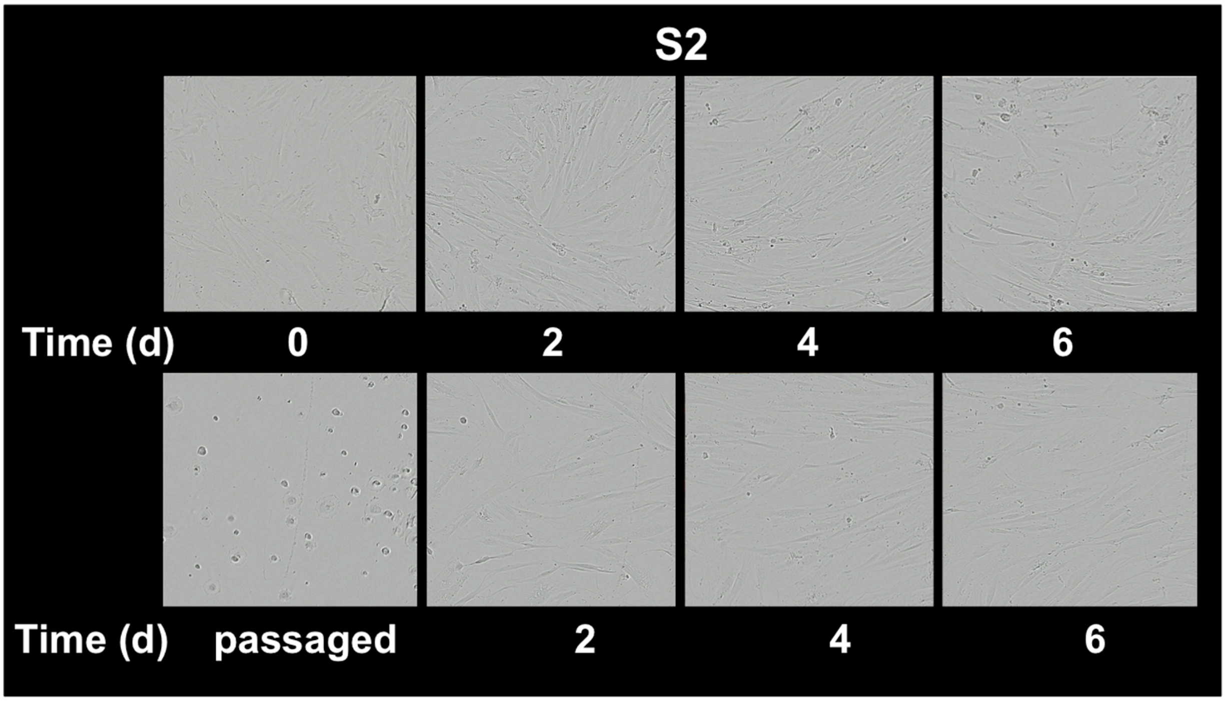

Appendix Figure S8

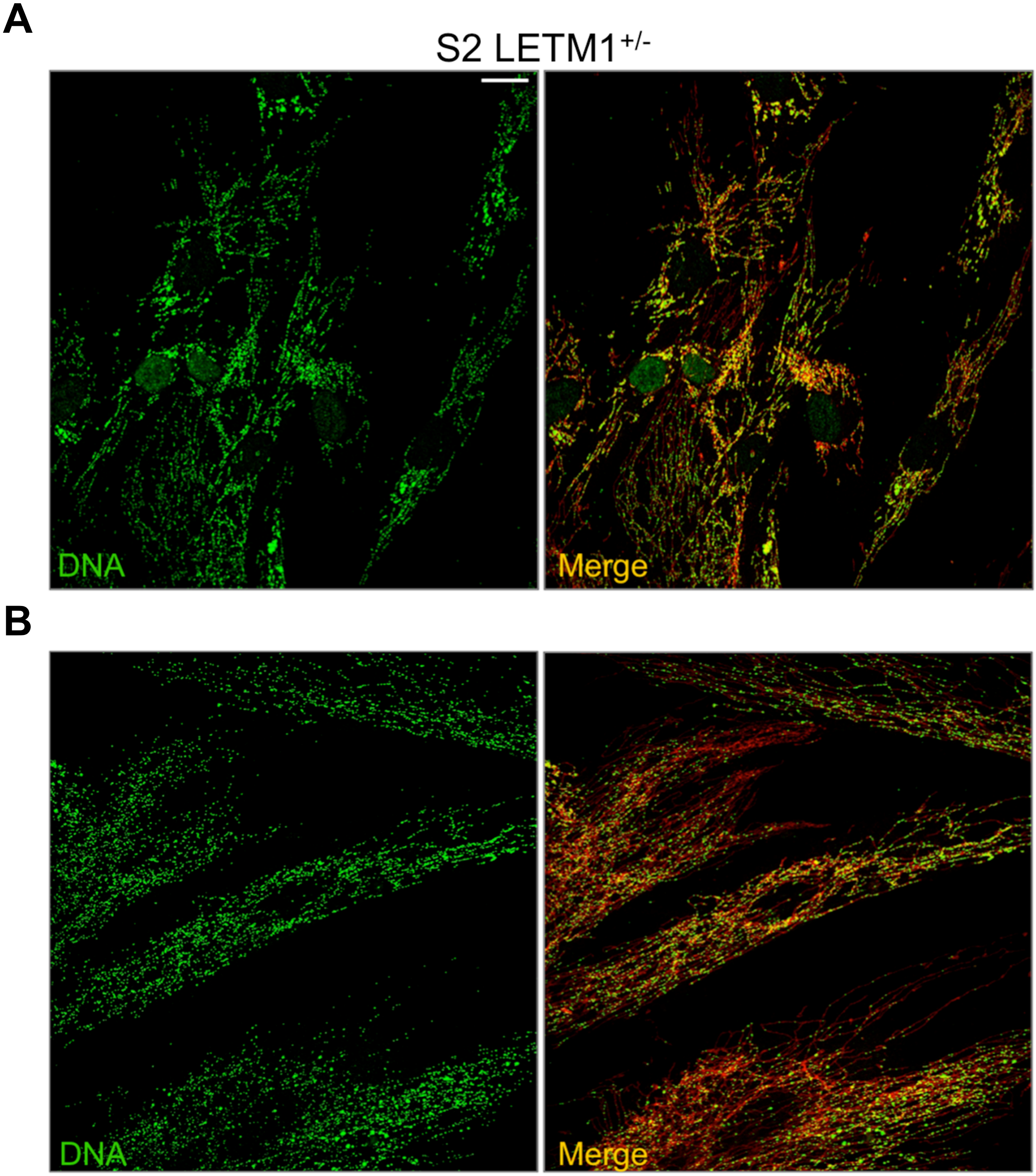

Appendix Figure S9

Appendix Table S1. List of the patients and associated deletions analyzed in this study

Patient	Deletion	Phenotype
WHS S1	arr 4p16.1p16.3(72447-6538014)x1 [6.5 Mb]	Severe growth delay, epilepsy, moderate to severe ID, typical facial dysmorphism
WHS S2	arr 4p15.32p16.3(72447-16899679)x1 [16.5 Mb]	Severe growth delay, epilepsy, severe ID, typical facial dysmorphism, microcephaly, hypospadias, scoliosis
WHS S3	arr 4p16.3(72447-2986280)x1 [3 Mb]	Severe growth delay, epilepsy, moderate to severe ID, typical facial dysmorphism, microcephaly, cleft palate
WHS S4	arr 4p16.1p16.3(72447-6440879)x1 [6.5 Mb]	Severe growth delay, epilepsy, severe ID, typical facial dysmorphism, microcephaly, atrial septal defect, pulmonary stenosis
WHS S5	arr 4p16.1p16.3(72447-1521506)x1 [1.5 Mb]	Febrile seizures as a child, borderline ID, partial facial dysmorphism

Appendix Table S2. List of all the primers employed throughout the study

Primer name	Sequence 5'-3'	Used for
β-2-globulin Forward	TCTTTTTCAGTGGGGGTGAA	
β-2-globulin Reverse	CTCACGTCATCCAGCAGAGA	
ATP6/8 Forward	CACAACACTAAAGGACGAACCT	
ATP6/8 Reverse	GGGATGGCCATGGCTAGGTTTA	
COX I Forward	GGAGCAGGAACAGGTTGAACAG	
COX I Reverse	GTTGTGATGAAATTGATGGC	
COX III Forward	TCCTCACTATCTGCTTCATCCG	

COX III Reverse	CCCTCATCAATAGATGGAGACA	qPCR
Cytb Forward	CTGATCCTCCAAATCACCACAG	
Cytb Reverse	GCGCCATTGGCGTGAAGGTA	
ND1 Forward	GAGCAGTAGCCCAAACAATCTC	
ND1 Reverse	GGGTCATGATGGCAGGAGTAAT	
12S Forward	AAACTGCTCGCCAGAACACT	
12S Reverse	CATGGGCTACACCTTGACCT	
16S Forward	GCTAAACCTAGCCCCAAACC	
16S Reverse	TTGGCTCTCCTTGCAAAGTT	
Hs-COXII-Forward	CGTCTGAACTATCCTGCCCG	
Hs-COXII-Reverse	TGGTAAGGGAGGGATCGTTG	
Hs-APP1 Forward	TTTTTGTGTGCTCTCCCAGGTCT	
Hs-APP1 Reverse	TGGTCACTGGTTGGTTGGC	

Appendix Table S3. List of all antibodies employed for Western Blot throughout the study

**Antibody**	**Company**	**Catalog ♯**	**Dilution**
anti-C7orf30	Abcam	Ab121631	1:500
anti-DPR1	Cell Signalling	8570	1:1000
anti-GAPDH	Santa Cruz	sc-11415	1:20 000
anti-LETM1 (M)	Abnova	H00003954-PW1	1:20 000
anti-MPV17	Proteintech	10310-1-AP	1:500
Anti-MPV17L2	Abcam	Ab81029	1:500
anti-MRPL11	Cell Signalling	2199	1:2000
anti-MRPL45	Proteintech	15682-1-AP	1:2000
anti-MRPS17	Proteintech	18881-1-AP	1:1000
anti-MRPS27	Proteintech	17280-1-AP	1:2000
Anti-MRPS29	Abcam	Ab11928	1:1000

anti-MTCO2	Abcam	Ab110258	1:1000
anti-NDUFB8	Abcam	Ab110242	1:2000
anti-PDH	Abcam	Ab110330	1:10000
anti-PDK3	Abcam	Ab55579	1:10000
anti-SER293-PDH	Abcam	Ab92696	1:1000
anti-SER616-DRP1	Cell Signalling	3455	1:1000
anti-TFAM	Gift from Professor Wiesner		1:4000
anti-TOM20	Santa Cruz	Sc-11415	1:30 000
anti-VINCULIN	Abcam	Ab14714	1:5000

Appendix Table S4. List of all antibodies employed for Immunofluorescence throughout the study

**Antibody**	**Company**	**Catalog ♯**	**Dilution**
AlexaFluor®-488 goat-anti mouse	Invitrogen	A10667	1:1000
AlexaFluor®-488 goat-anti rat	Invitrogen	A11006	1:1000
AlexaFluor®-568 donkey anti rabbit	Invitrogen	A10042	1:1000
anti-BrdU	Thermo Fisher	B35128	1:200
anti-BrdU	BioRad	MCA2060	1:200
anti-DNA	Progen	61014	1:300
anti-LETM1 (M)	Abnova	H00003954-PW1	1:200
anti-LETM1 (R)	Proteintech	16024-1-AP	1:200
anti-MRPL45	Proteintech	15682-1-AP	1:200
anti-MRPS18B	Proteintech	16139-1-AP	1:200
anti-TOM20	Abcam	Ab186735	1:600 (Fibroblasts) or 1:400 (HeLa)

Appendix Table S5: Statistical analysis

Fig 1B

COXI,ND4

One Way ANOVA multiple comparisons test	Mean Diff.	95.00% CI of diff.	Significant?	Summary	Adjusted P Value
NT vs. siR1+siR3	0.4988	0.2919 to 0.7057	Yes	***	<0.001
NT vs. siR2	0.07185	-0.1351 to 0.2788	No	ns	0.6285

Cytb,ND2

One Way ANOVA multiple comparisons test	Mean Diff.	95.00% CI of diff.	Significant?	Summary	Adjusted P Value
NT vs. siR1+siR3	0.698	0.4965 to 0.8995	Yes	***	<0.001
NT vs. siR2	0.2891	0.08758 to 0.4906	Yes	**	0.005

COIII,COII

One Way ANOVA multiple comparisons test	Mean Diff.	95.00% CI of diff.	Significant?	Summary	Adjusted P Value
NT vs. siR1+siR3	0.6895	0.5552 to 0.8239	Yes	***	<0.001
NT vs. siR2	0.2137	0.0794 to 0.3481	Yes	**	0.002

ATP6

One Way ANOVA multiple comparisons test	Mean Diff.	95.00% CI of diff.	Significant?	Summary	Adjusted P Value
NT vs. siR1+siR3	0.5716	0.3313 to 0.8118	Yes	***	<0.001
NT vs. siR2	0.04747	-0.1928 to 0.2877	No	ns	0.8544

ND6,ND3

One Way ANOVA multiple comparisons test	Mean Diff.	95.00% CI of diff.	Significant?	Summary	Adjusted P Value
NT vs. siR1+siR3	0.7088	0.4946 to 0.923	Yes	***	<0.001
NT vs. siR2	0.1279	-0.08627 to 0.3421	No	ns	0.284

ND4L,ATP8

One Way ANOVA multiple comparisons test	Mean Diff.	95.00% CI of diff.	Significant?	Summary	Adjusted P Value
NT vs. siR1+siR3	0.6073	0.1378 to 1.077	Yes	*	0.011
NT vs. siR2	-0.01657	-0.486 to 0.4529	No	ns	0.994

Fig 1D

LETM1 KD (> 50%)	**LETM1**
vs.	vs
NT	**LETM1**
Unpaired t test with Welch's correction	
P value	0.02
P value summary	*
Significantly different (P < 0.05)?	Yes
One- or two-tailed P value?	Two-tailed
Welch-corrected t, df	t=6.9 df=2

LETM1 KD (< 50%)	**LETM1**
vs.	vs
NT	**LETM1**
Unpaired t test with Welch's correction	
P value	<0.001
P value summary	***
Significantly different (P < 0.05)?	Yes
One- or two-tailed P value?	Two-tailed
Welch-corrected t, df	t=20.24 df=14

LETM1 KD (> 50%)	**NDUFB8**
vs.	vs
NT	**NDUFB8**
Unpaired t test with Welch's correction	
P value	0.002
P value summary	**
Significantly different (P < 0.05)?	Yes
One- or two-tailed P value?	Two-tailed
Welch-corrected t, df	t=6.978 df=4

LETM1 KD (< 50%)	**NDUFB8**
vs.	vs
NT	**NDUFB8**
Unpaired t test with Welch's correction	
P value	<0.001
P value summary	***
Significantly different (P < 0.05)?	Yes
One- or two-tailed P value?	Two-tailed
Welch-corrected t, df	t=8.457 df=7

LETM1 KD (> 50%)	**COX II**
vs.	vs
NT	**COX II**

Unpaired t test with Welch's correction	
P value	<0.001
P value summary	***
Significantly different (P < 0.05)?	Yes
One- or two-tailed P value?	Two-tailed
Welch-corrected t, df	t=8.874 df=5

LETM1 KD (< 50%)	**COX II**
vs.	vs
NT	**COX II**

Unpaired t test with Welch's correction	
P value	<0.001
P value summary	***
Significantly different (P < 0.05)?	Yes
One- or two-tailed P value?	Two-tailed
Welch-corrected t, df	t=11.64 df=13

Fig 1E

One Way ANOVA	Mean Diff.	95.00% CI of diff.	Significant?	Summary	Adjusted P Value
**BASAL Respiration**					
NT vs. siR1	0.4019	0.02937 to 0.7744	Yes	*	0.029
NT vs. siR2	0.2576	-0.1149 to 0.6301	No	ns	0.321
**MAXIMAL Respiration**					
NT vs. siR1	0.7415	0.369 to 1.114	Yes	***	<0.001
NT vs. siR2	0.4983	0.1258 to 0.8708	Yes	**	0.004

Fig 2A

One Way ANOVA multiple comparisons test	Mean Diff.	95.00% CI of diff.	Significant?	Summary	Adjusted P Value
**MRPL11**					
NT vs. siR1	0.5107	0.3433 to 0.678	Yes	***	<0.001
NT vs. siR2	0.17	0.002676 to 0.3373	Yes	*	0.047

One Way ANOVA multiple comparisons test	Mean Diff.	95.00% CI of diff.	Significant?	Summary	Adjusted P Value
**C7ORF30**					
NT vs. siR1	0.4733	0.2314 to 0.7152	Yes	**	0.002
NT vs. siR2	0.5437	0.3018 to 0.7856	Yes	**	0.001

One Way ANOVA multiple comparisons test	Mean Diff.	95.00% CI of diff.	Significant?	Summary	Adjusted P Value

**MRPS17**					
NT vs. siR1	0.4933	0.2049 to 0.7817	Yes	**	0.003
NT vs. siR2	0.4628	0.1958 to 0.7297	Yes	**	0.003

Fig 2B

One Way ANOVA multiple comparisons test	Mean Diff.	95.00% CI of diff.	Significant?	Summary	Adjusted P Value
**12S**					
NT vs. siR1	0.511	0.2713 to 0.7508	Yes	***	<0.001
NT vs. siR2	0.1992	-0.04057 to 0.439	No	ns	0.115
NT vs. siR3	0.3193	0.05121 to 0.5873	Yes	*	0.018
One Way ANOVA multiple comparisons test					
**16S**					
NT vs. siR1	0.3748	0.09611 to 0.6536	Yes	**	0.007
NT vs. siR2	0.08186	-0.1969 to 0.3606	No	ns	0.794
NT vs. siR3	0.04731	-0.2643 to 0.3589	No	ns	0.961

Fig 2C

**MRPS**	
siR1	**Fraction 3**
vs.	vs.
NT	**Fraction 3**
Unpaired t test with Welch's correction	
P value	0.03
P value summary	*
Significantly different (P < 0.05)?	Yes
One- or two-tailed P value?	Two-tailed
Welch-corrected t, df	t=3.849 df=3.009
siR1	**Fraction 11**
vs.	vs.
NT	**Fraction 11**
Unpaired t test with Welch's correction	
P value	0.029
P value summary	*
Significantly different (P < 0.05)?	Yes
One- or two-tailed P value?	Two-tailed
Welch-corrected t, df	t=5.712 df=2.002
siR1	**Fraction 12**
vs.	vs.
NT	**Fraction 12**

Unpaired t test with Welch's correction	
P value	0.016
P value summary	*
Significantly different (P < 0.05)?	Yes
One- or two-tailed P value?	Two-tailed
Welch-corrected t, df	t=4.309 df=3.559

MRPL

siR1	**Fraction 2**
vs.	vs.
NT	**Fraction 2**

Unpaired t test with Welch's correction	
P value	0.023
P value summary	*
Significantly different (P < 0.05)?	Yes
One- or two-tailed P value?	Two-tailed
Welch-corrected t, df	t=5.863 df=2.159

Fig 3A

One Way ANOVA multiple comparisons test	Mean Diff.	95.00% CI of diff.	Significant?	Summary	Adjusted P Value
**mtDNA copy number**					
NT vs. siR1	-0.6491	-1.287 to -0.01122	Yes	*	0.046
NT vs. siR2	-0.9238	-1.562 to -0.2859	Yes	*	0.01

One Way ANOVA multiple comparisons test	Mean Diff.	95.00% CI of diff.	Significant?	Summary	Adjusted P Value
**RNA Levels**					
NT vs. siR1	0.354	0.1908 to 0.5172	Yes	***	<0.001
NT vs. siR2	0.1531	-0.01012 to 0.3163	No	ns	0.071
NT vs. siR3	-0.1902	-0.3727 to -0.00774	Yes	*	0.038

Fig 4A

One Way ANOVA multiple comparisons test	Mean Diff.	95.00% CI of diff.	Significant?	Summary	Adjusted P Value
**% Cell abnormal mtDNA**					
NT vs. siR1	-66	-82.04 to -49.96	Yes	***	<0.001
NT vs. siR2	-37.45	-54.46 to -20.44	Yes	***	<0.001
NT vs. siR3	-90.4	-106.4 to -74.36	Yes	***	<0.001

Fig 6A

One Way ANOVA multiple comparisons test	Mean Diff.	95.00% CI of diff.	Significant?	Summary	Adjusted P Value
**DRP1**					

NT vs. siR1	0.1452	-0.05552 to 0.3458	No	ns	0.185
NT vs. siR2	0.1806	-0.01077 to 0.3719	No	ns	0.066
NT vs. siR3	0.3896	0.1553 to 0.624	Yes	**	0.001

One Way ANOVA multiple comparisons test	Mean Diff.	95.00% CI of diff.	Significant?	Summary	Adjusted P Value
**P-DRP1**					
NT vs. siR1	-1.267	-1.971 to -0.5622	Yes	**	0.001
NT vs. siR2	0.2627	-0.4418 to 0.9672	No	ns	0.63

Fig 6C

One Way ANOVA multiple comparisons test	Mean Diff.	95.00% CI of diff.	Significant?	Summary	Adjusted P Value
**OXPHOS**					
NT vs. siR1	0.8745	0.6012 to 1.148	Yes	***	<0.001
NT vs. siD	0.1438	-0.1295 to 0.4171	No	ns	0.569
NT vs. siR1D	0.4776	0.2043 to 0.7509	Yes	***	<0.001
siR1 vs. siR1D	-0.3969	-0.6702 to -0.1236	Yes	**	0.002
siD vs. siR1D	0.3337	0.06042 to 0.607	Yes	*	0.011

Fig 8A

One Way ANOVA multiple comparisons test	Mean Diff.	95.00% CI of diff.	Significant?	Summary	Adjusted P Value
**LETM1 Levels**					
Ctrls vs. S1	37.87	21.09 to 54.65	Yes	***	<0.001
Ctrls vs. S2	31.94	11.67 to 52.21	Yes	**	0.001
Ctrls vs. S3	29.52	11.36 to 47.69	Yes	***	<0.001
Ctrls vs. S4	40.77	22.61 to 58.94	Yes	***	<0.001
Ctrls vs. S5	6.523	-11.64 to 24.69	No	ns	0.796

Fig 10C

One Way ANOVA multiple comparisons test	Mean Diff.	95.00% CI of diff.	Significant?	Summary	Adjusted P Value
**% Cell with mtDNA aggregation**					
Ctrls+S5 vs. S1	-25.75	-37.391076 to -14.108924	Yes	***	<0.001
Ctrls+S5 vs. S4	-26.666667	-39.418827 to -13.914507	Yes	***	<0.001
Ctrls+S5 vs. S2	-59	-71.75216 to -46.24784	Yes	***	<0.001
Ctrls+S5 vs. S3	-68.333333	-81.085493 to -55.581173	Yes	***	<0.001

Fig 10D

One Way ANOVA multiple comparisons test	Mean Diff.	95.00% CI of diff.	Significant?	Summary	Adjusted P Value
**mtDNA copy number**					
Ctrls vs. S1 +S4	-1.036	-1.679 to -0.3941	Yes	**	0.004
Ctrls vs. S5	-0.03708	-0.7364 to 0.6623	No	ns	0.986

Fig 10E

One Way ANOVA multiple comparisons test	Mean Diff.	95.00% CI of diff.	Significant?	Summary	Adjusted P Value
**DRP1**					
Ctrls vs. $WHS^{+/-}$	0.2616	0.06063 to 0.4626	Yes	**	0.009
Ctrls vs. $WHS^{+/+}$	0.1789	-0.1029 to 0.4607	No	ns	0.256

One Way ANOVA multiple comparisons test	Mean Diff.	95.00% CI of diff.	Significant?	Summary	Adjusted P Value
**DRP1616/Total DRP1**					
Ctrls vs. $WHS^{+/-}$	0.6088	0.3775 to 0.84	Yes	***	<0.001
Ctrls vs. $WHS^{+/+}$	-0.08333	-0.3916 to 0.225	No	ns	0.738

Fig 11C

$WHS^{+/-}$	**PDH**
vs.	vs
Ctrls	**PDH**
Unpaired t test with Welch's correction	
P value	0.627
P value summary	ns
Significantly different (P < 0.05)?	No
One- or two-tailed P value?	Two-tailed
Welch-corrected t, df	t=0.5063 df=7.545

$WHS^{+/-}$	**PDH/P-PDH**
vs.	vs
Ctrls	**PDH/P-PDH**
Unpaired t test with Welch's correction	
P value	0.011
P value summary	*
Significantly different (P < 0.05)?	Yes
One- or two-tailed P value?	Two-tailed
Welch-corrected t, df	t=3.543 df=6.146

One Way ANOVA multiple comparisons test	Mean Diff.	95.00% CI of diff.	Significant?	Summary	Adjusted P Value
**PDK3**					
Ctrls vs. $WHS^{+/-}$	-1.635	-2.764 to -0.5063	Yes	**	0.006
Ctrls vs. $WHS^{+/+}$	0.3017	-1.017 to 1.62	No	ns	0.79
