# Supplementary material for: LETM1 couples mitochondrial DNA metabolism and nutrient preference
Source: EMBO Mol Med. 2018 Jul 16;10(9):e8550. doi: 10.15252/emmm.201708550 (PMC6127893; doi:10.15252/emmm.201708550)
Supplement: Supplementary file 2 — Expanded View Figures PDF [file EMMM-10-e8550-s002.pdf]

Expanded View Figures

Figure EV1. *LETM1* repression perturbs mtDNA organization.

A Further examples of the mtDNA abnormalities are shown in Fig 3A. *LETM1* expression was suppressed in HeLa cells by transfection with targeted si*RNAs* (siR1, siR2, or siR3). A non-target dsRNA (NT) served as control. Cells were fixed and immunolabeled with anti-DNA antibody (green). Scale bar: 15 μm.

B HeLa cells stained with anti-LETM1 antibody (red) and anti-BrdU (green) after labeling with 5 mM BrU for 60 min. In other images, LETM1 was stained green and other proteins stained red using antibodies to the RNA granule protein GRSF1, the 55S ribosome component MRPL45, or the outer mitochondrial membrane protein TOM20. Scale bars are 12 μm in the main images and 3 μm in offset magnification.

© 2018 The Authors

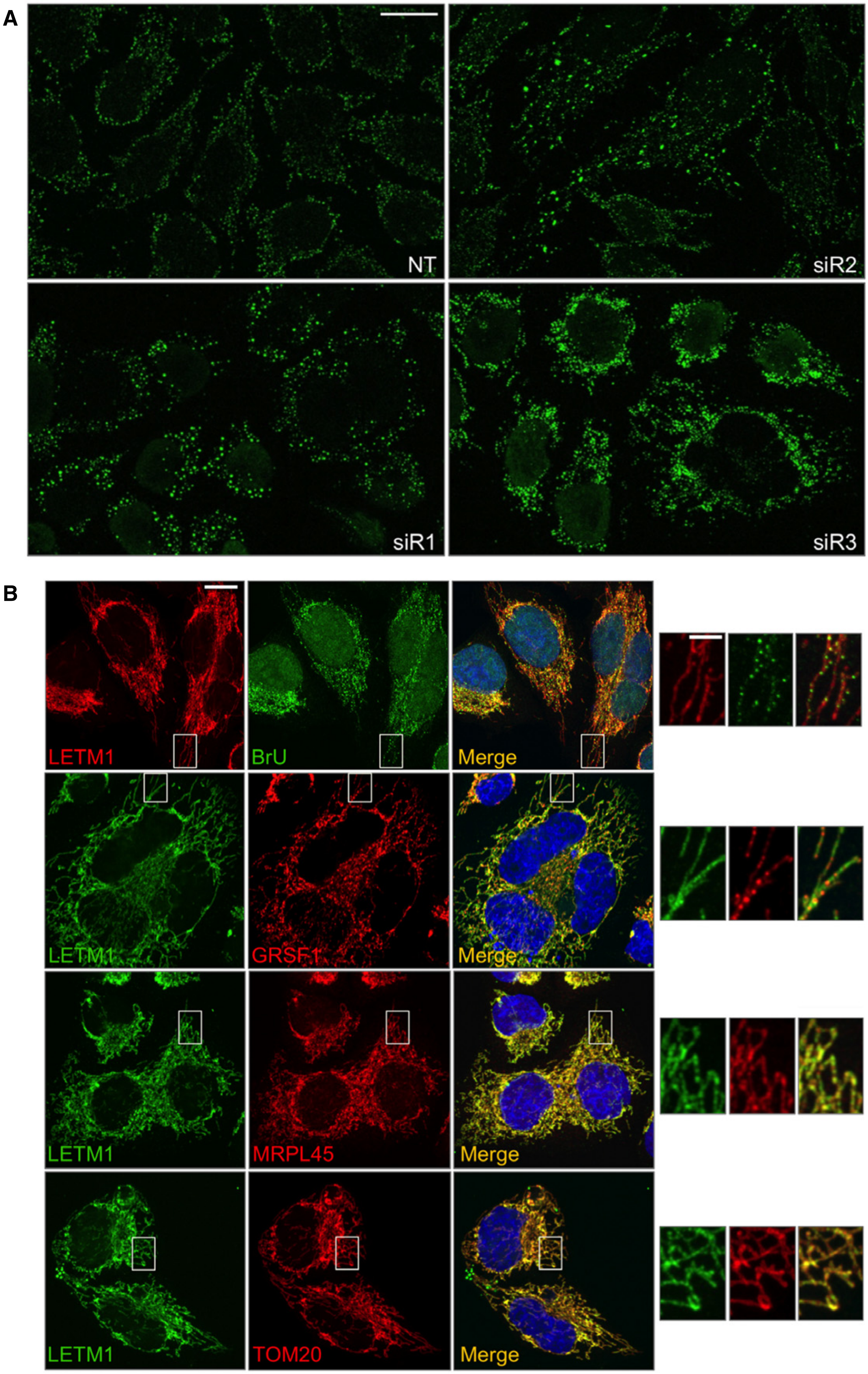

Figure EV1.

© 2018 The Authors

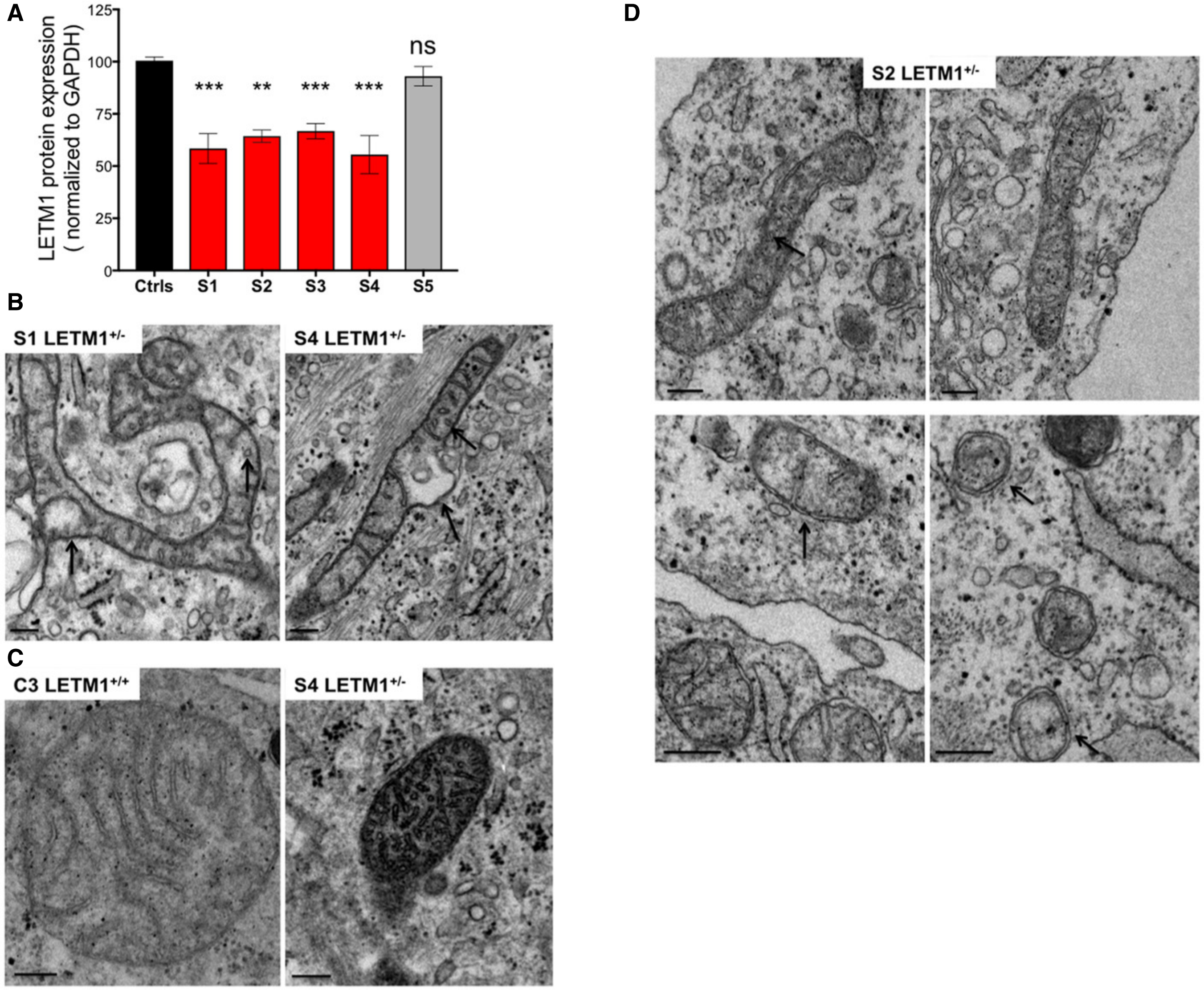

Figure EV2. ***LETM1*** **haploinsufficiency in WHS is associated with mitochondrial ultrastructure abnormalities.**

A Quantification of LETM1 expression in fibroblasts. Data from control cell lines (ctrls) C1–C3 were pooled. All data are expressed as mean ± SEM (n = 4 or 5). $**P < 0.01$, $***P < 0.001$. ns, not statistically significant (one-way ANOVA).

B Magnification of WHS *LETM1*$^{+/-}$ S1 and S4 mitochondria. Black arrows indicate circular cristae and matrix distensions. Scale bar: 0.2 μm.

C Magnification of control C3 and WHS *LETM1*$^{+/-}$ S4 mitochondria showing dense matrix and circular cristae exclusively in the latter. Scale bar: 0.2 μm.

D Electron micrographs of WHS *LETM1*$^{+/-}$ (S2) fibroblasts. Black arrows indicate circular cristae in the upper 2 images and mitochondria with sparse cristae in the lower images. Scale bar: 0.2 μm.

Figure EV3. **PDH is repressed in WHS *LETM1*$^{+/-}$ fibroblasts, and unlike controls cells, they are able to grow on medium containing KB in place of glucose and pyruvate.**

A Quantification of total PDH, PDH^{S293}, and PDK3 immunoblots. Data are expressed as mean ± SEM of n = 5 independent experiments. $*P < 0.05$, $**P < 0.01$. ns, not statistically significant; *t*-test with Welch's correction for PDH and PDH^{S293}; one-way ANOVA for PDK3.

B Light microscope images of control (C2), WHS *LETM1*$^{+/+}$ (S5), and WHS *LETM1*$^{+/-}$ (S1, S3, and S4) cells growing in 0.3 mM BHB (KB) for 6 days, after a switch from 25 mM glucose and 1 mM pyruvate at 40% confluence. Scale bar: 300 μm.

C S4 cells grown to confluency in KB medium (as per panel A) were passaged and cultured for a further 6 days.

© 2018 The Authors

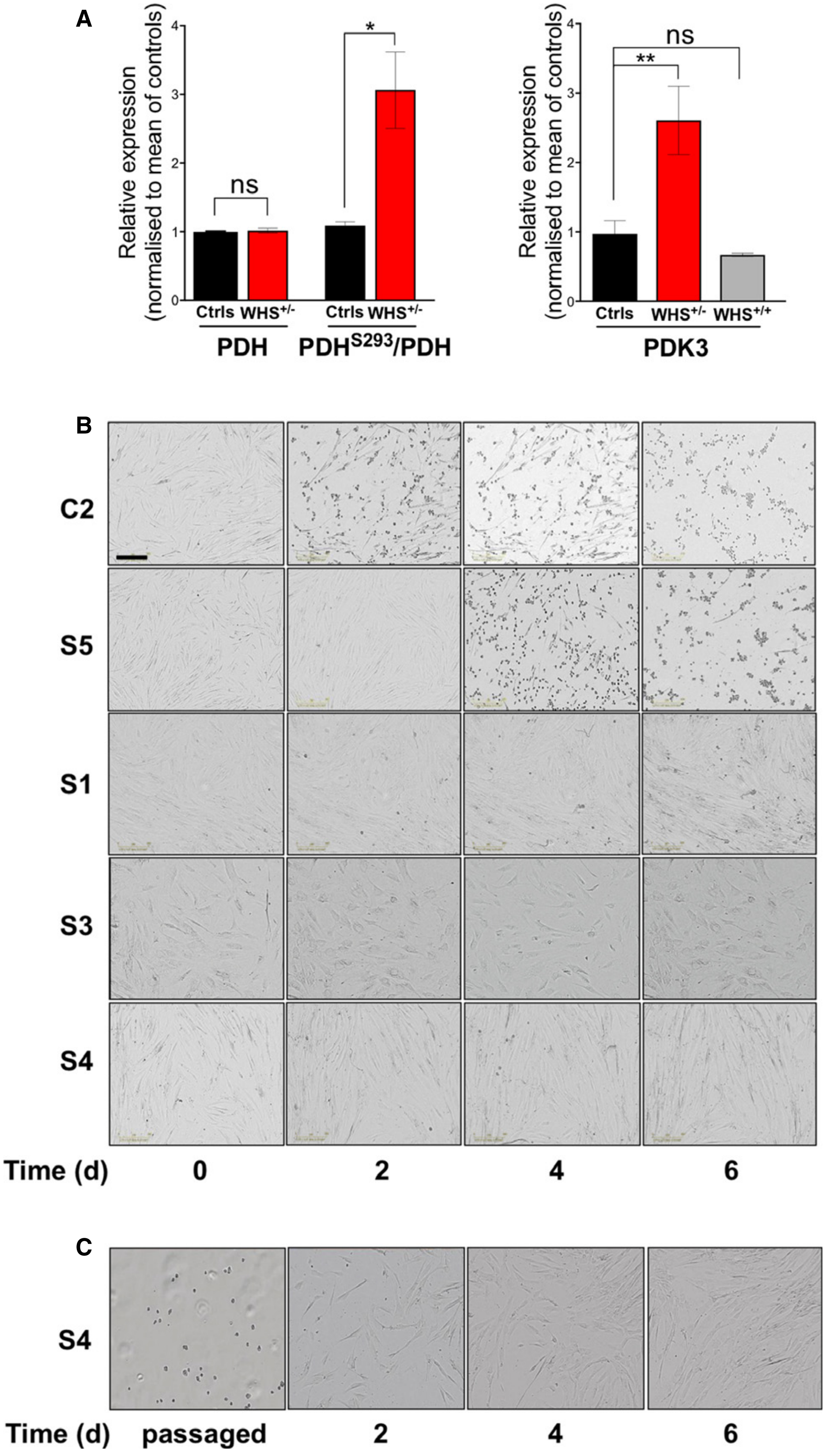

Figure EV3.

 © 2018 The Authors
