# Supplementary material for: LETM1 couples mitochondrial DNA metabolism and nutrient preference
Source: EMBO Mol Med. 2018 Jul 16;10(9):e8550. doi: 10.15252/emmm.201708550 (PMC6127893; doi:10.15252/emmm.201708550)
Supplement: Supplementary file 5 — Movie Legends [file EMMM-10-e8550-s005.pdf]

Movie EV1. Control fibroblasts (C1) do not survive when ketone bodies replace glucose in the growth medium. The video starts at day 0, 24 hours after cells were plated. At Day 1, standard DMEM growth medium is replaced with BHB supplemented medium (see Materials and Methods)**.** The effect on cell viability is recorded for 7 days.

Movie EV2. WHS (S4) derived fibroblasts survive when ketone bodies replace glucose in the growth medium. The video starts at day 0, 24 hours after cells were plated. At Day 1, standard DMEM growth medium is replaced with BHB supplemented medium (see Materials and Methods)**.** The effect on cell viability is recorded for 7 days.
