# Supplementary figures and images for: LETM1 couples mitochondrial DNA metabolism and nutrient preference
Source: EMBO Mol Med. 2018 Jul 16;10(9):e8550. doi: 10.15252/emmm.201708550 (PMC6127893; doi:10.15252/emmm.201708550)

RAW DATA Figure 1B

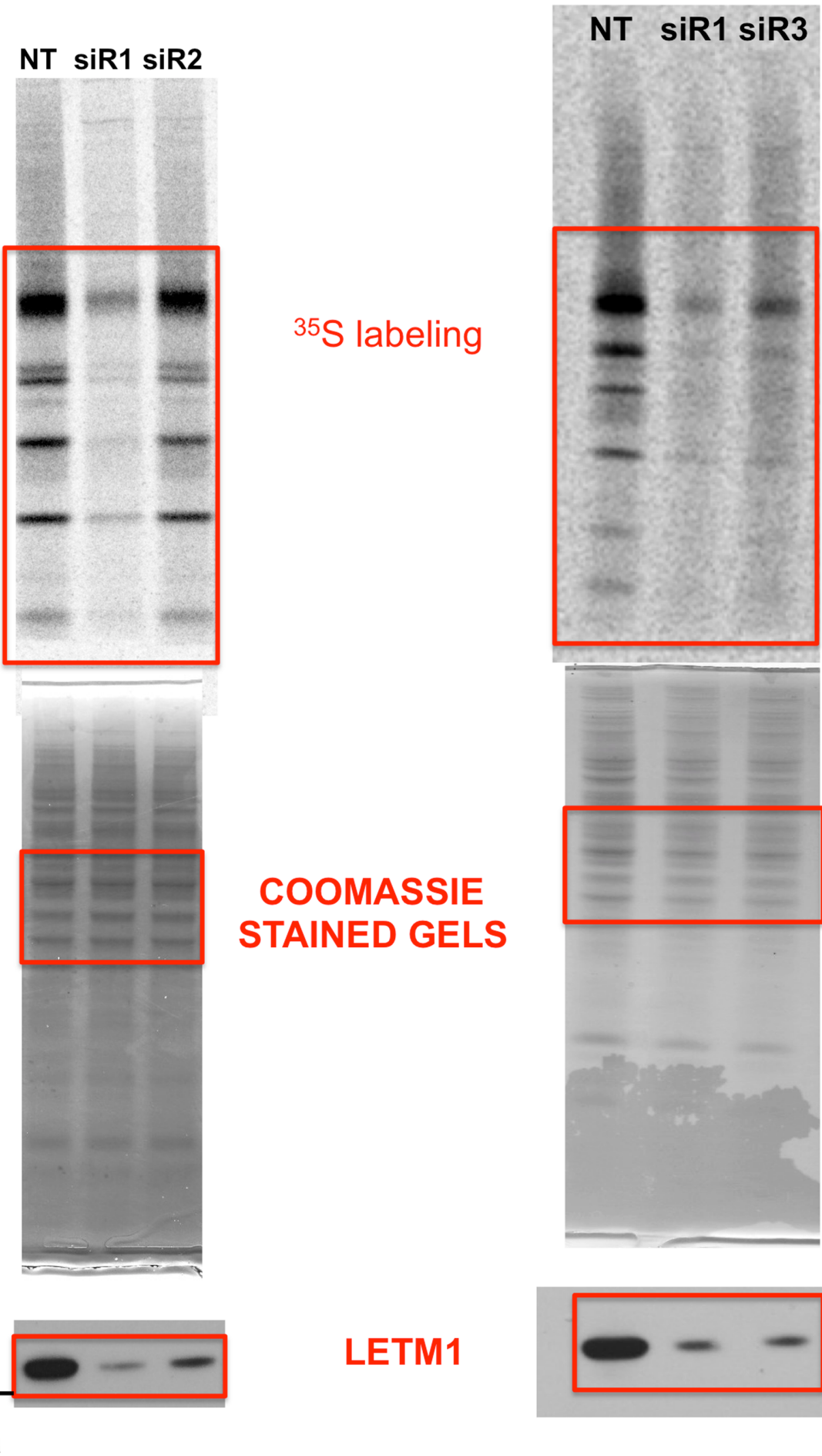

# RAW DATA Figure 1D

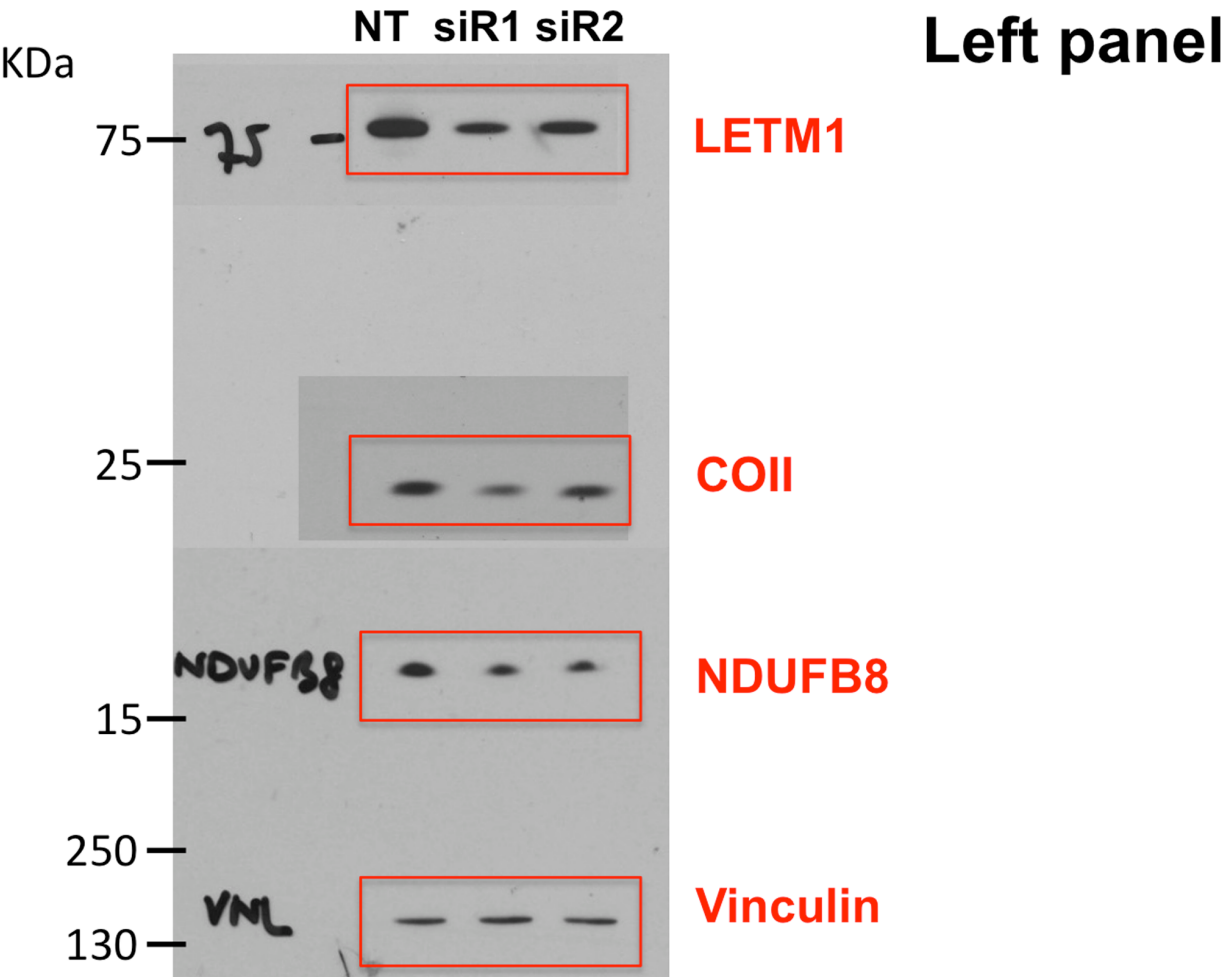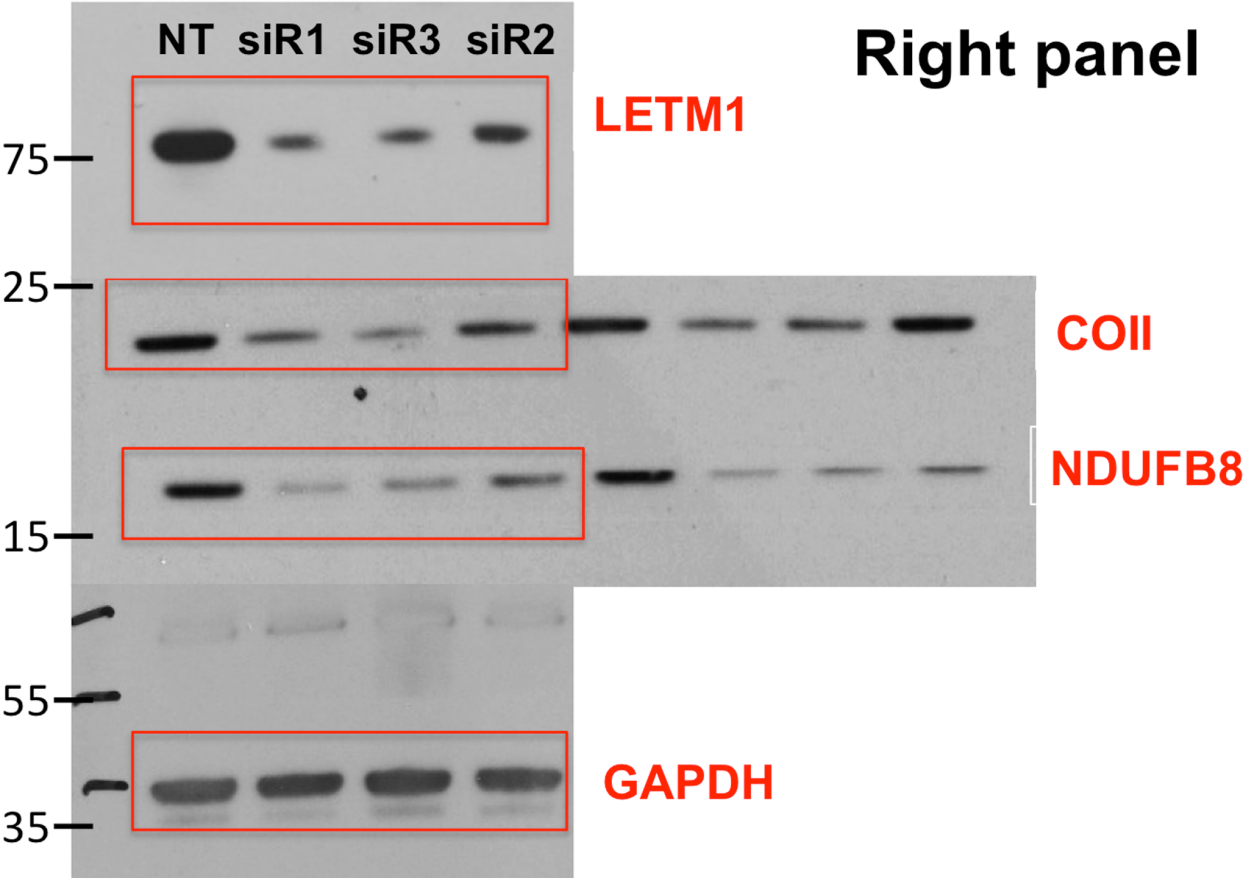

Supplement: Supplementary file 7 — Source Data for Figure 1 [file EMMM-10-e8550-s006.pdf]

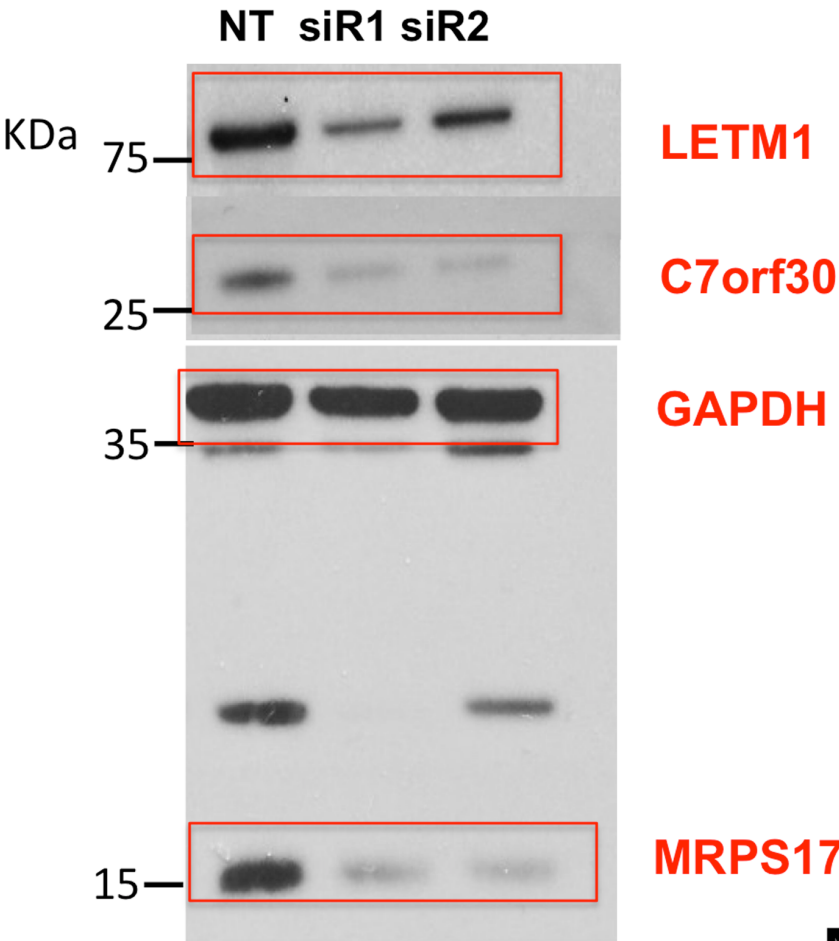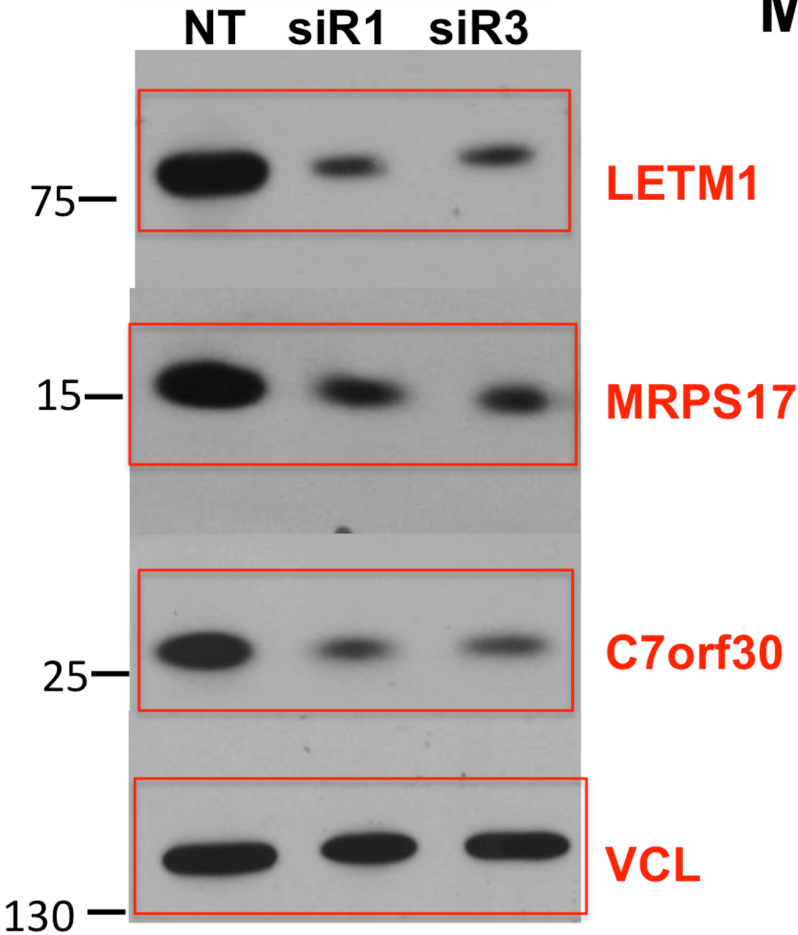

# RAW DATA Figure 2A

Right panel

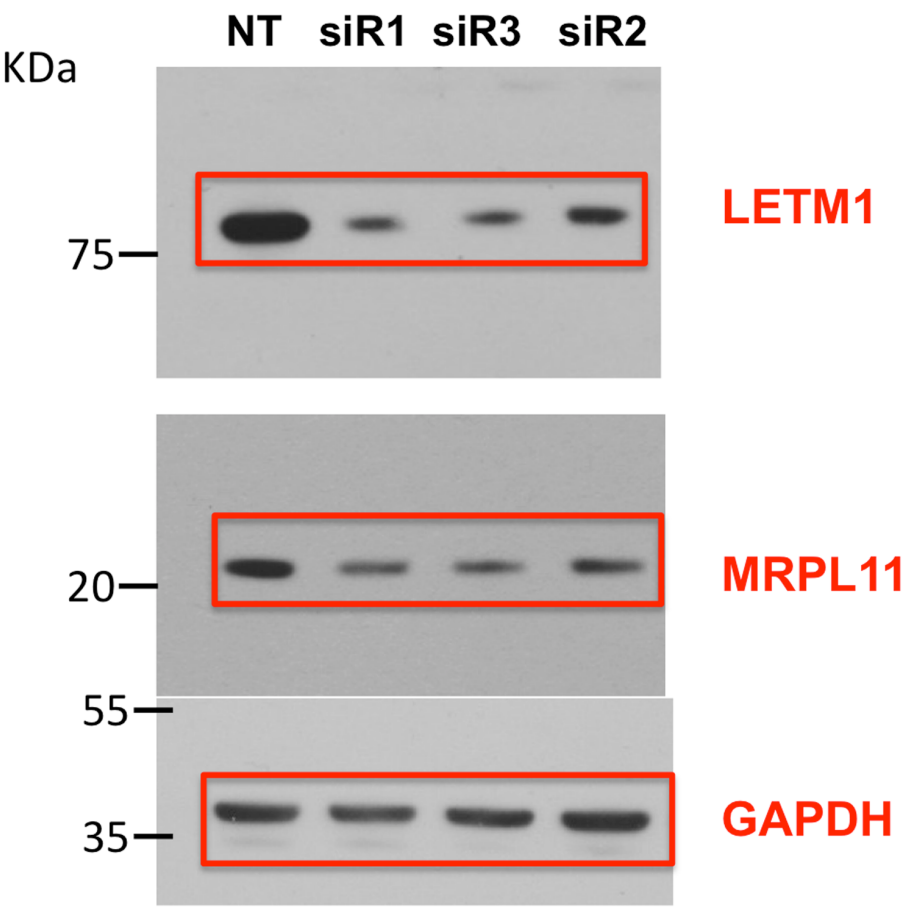

# RAW DATA Figure 2C

KDa

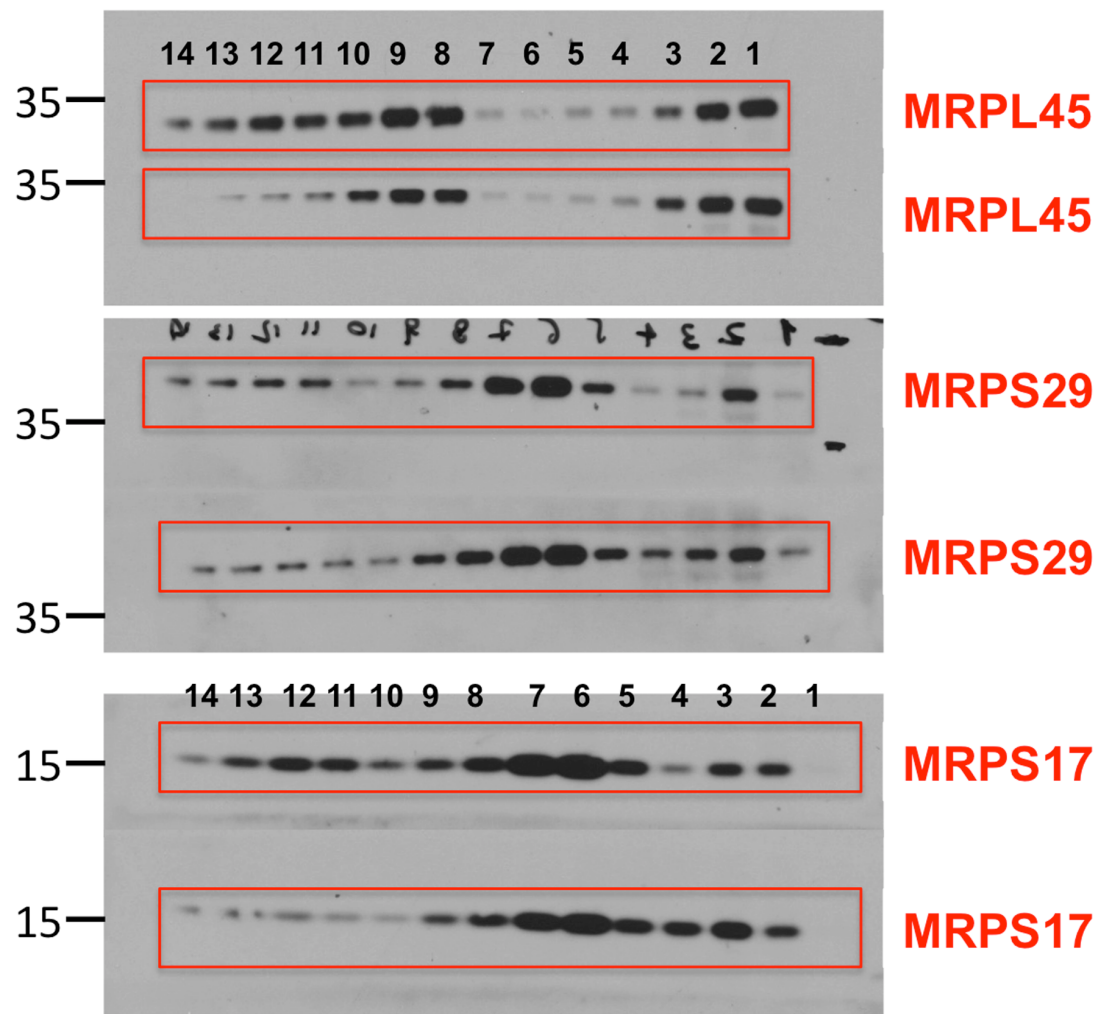

Supplement: Supplementary file 8 — Source Data for Figure 2 [file EMMM-10-e8550-s007.pdf]

# RAW DATA Figure 4C

KDa

4 5 6 7 8 9 10 11 12 13 14 15

75

# LETM1

25

# TEAM

20

# MRPL11

15

MRPS17

25

## C7orf30

15

MPV17

25

# MPV17L2

# RAW DATA Figure 4D

KDa

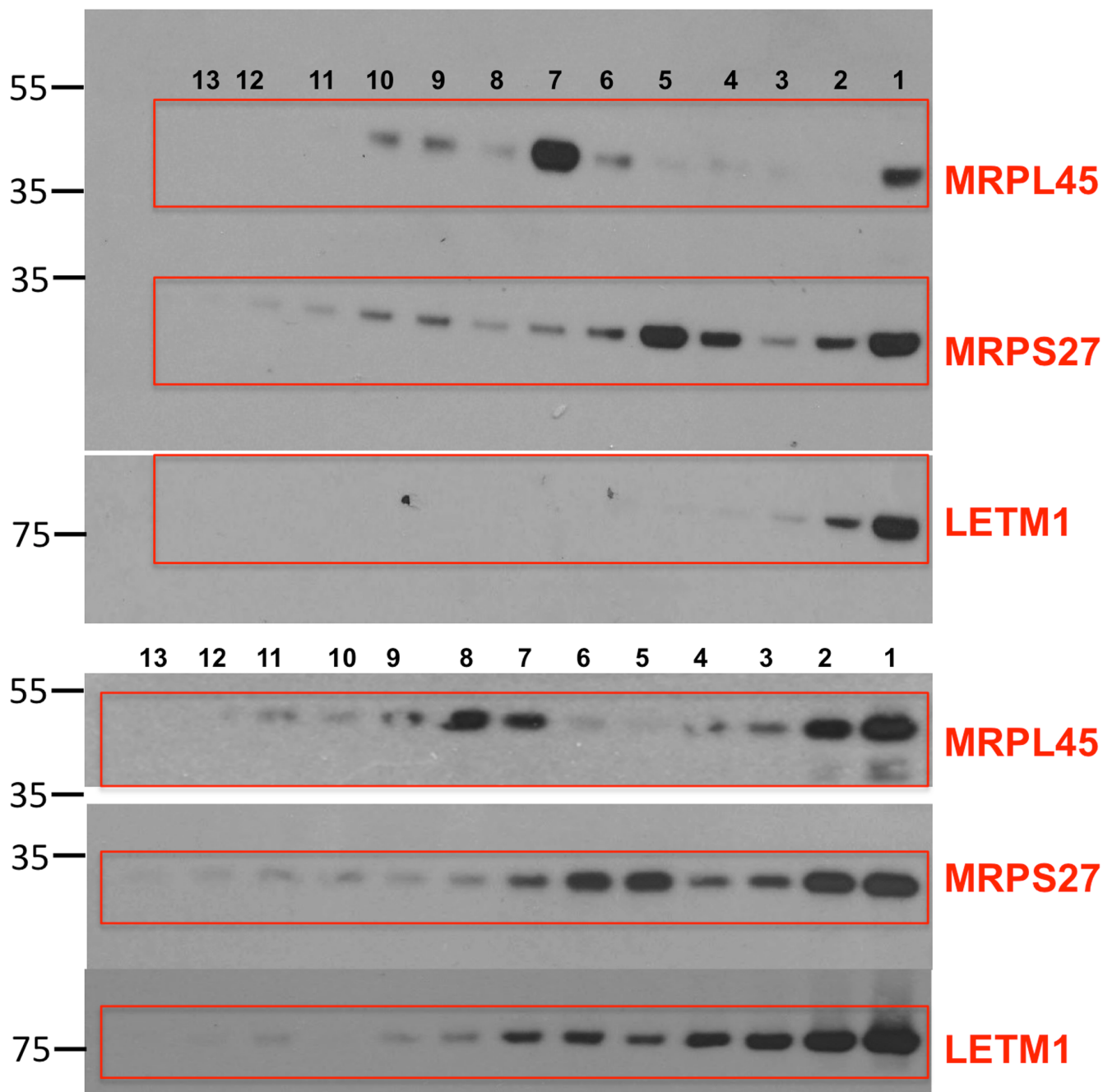

Supplement: Supplementary file 10 — Source Data for Figure 4 [file EMMM-10-e8550-s009.pdf]

# RAW DATA Figure 5 A

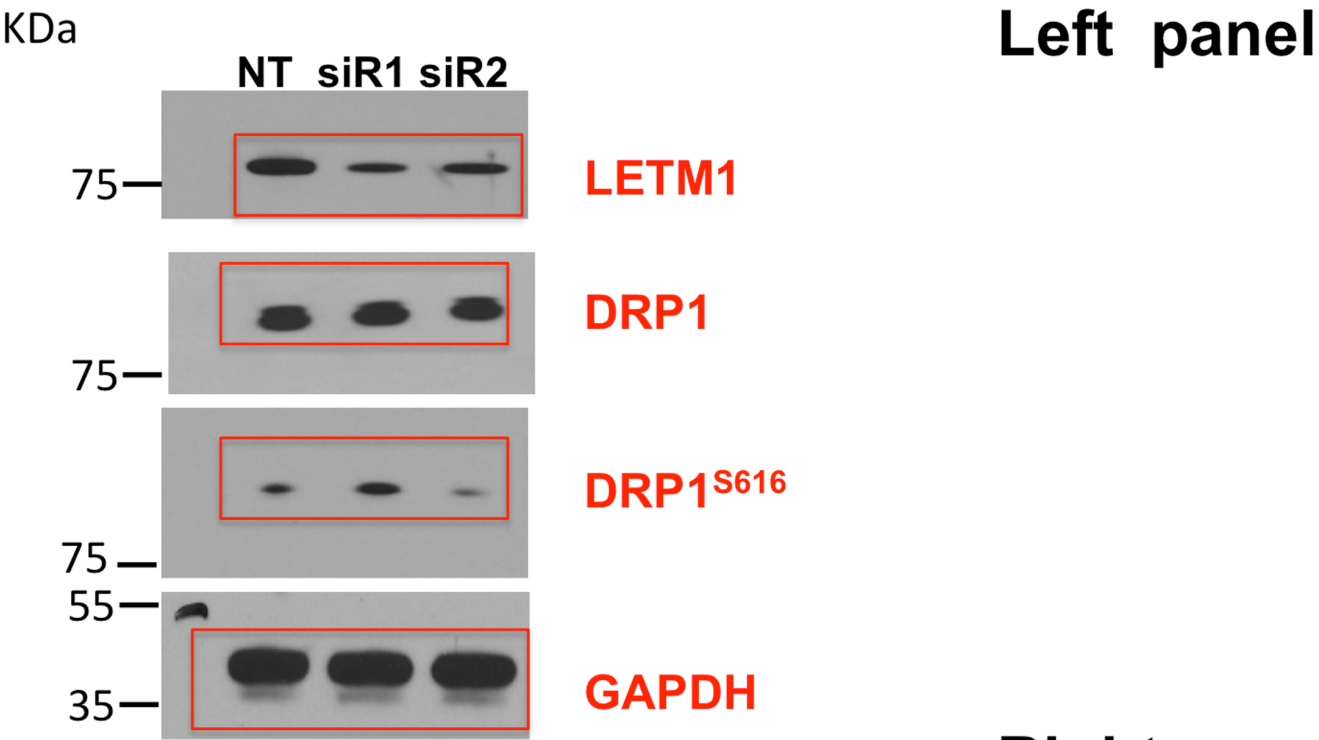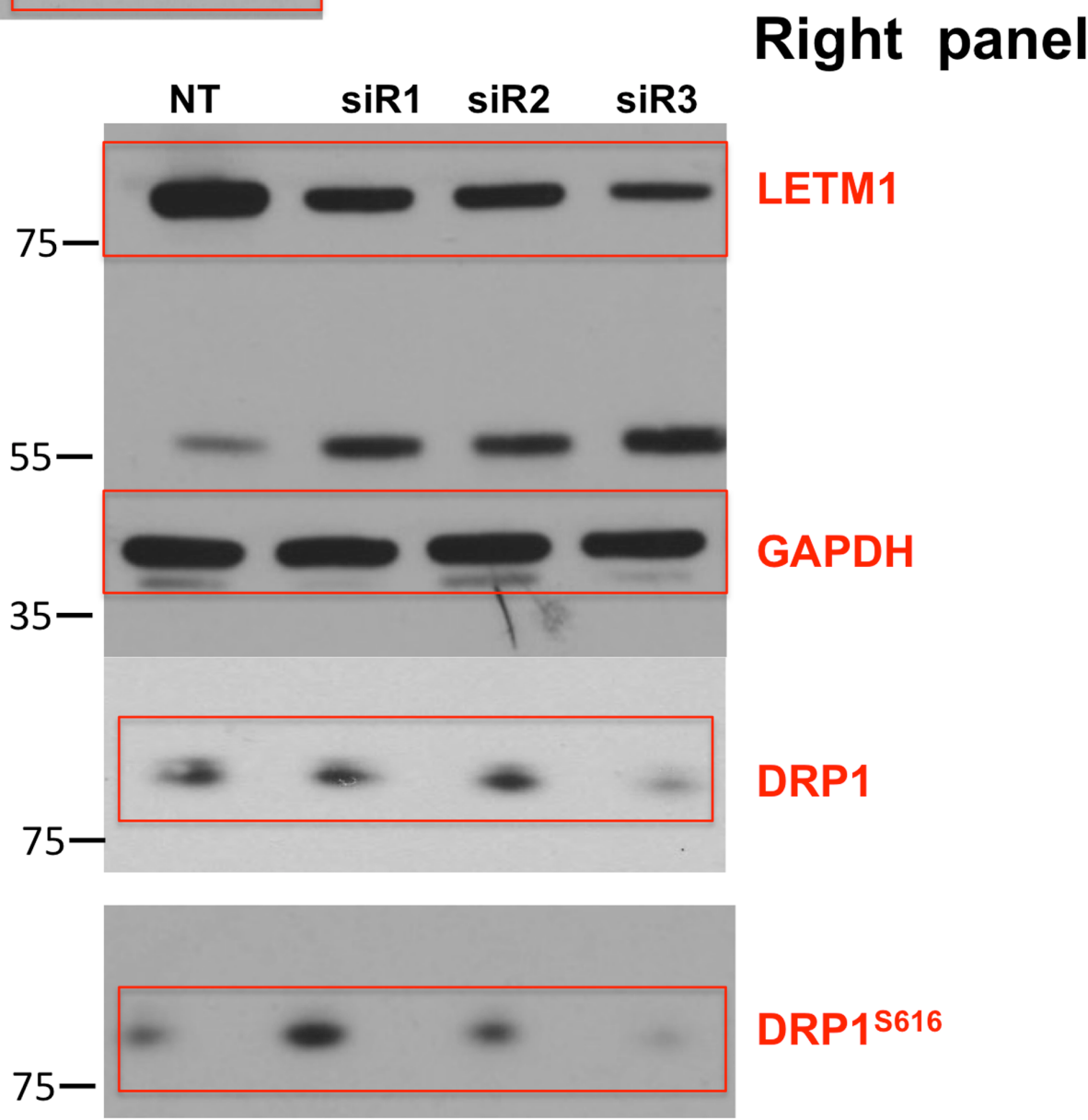

# RAW DATA Figure 5B

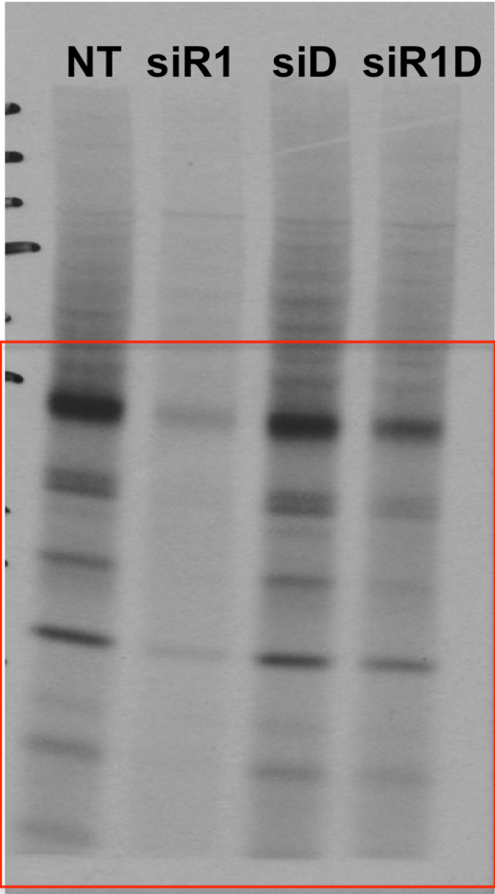

35S labeling

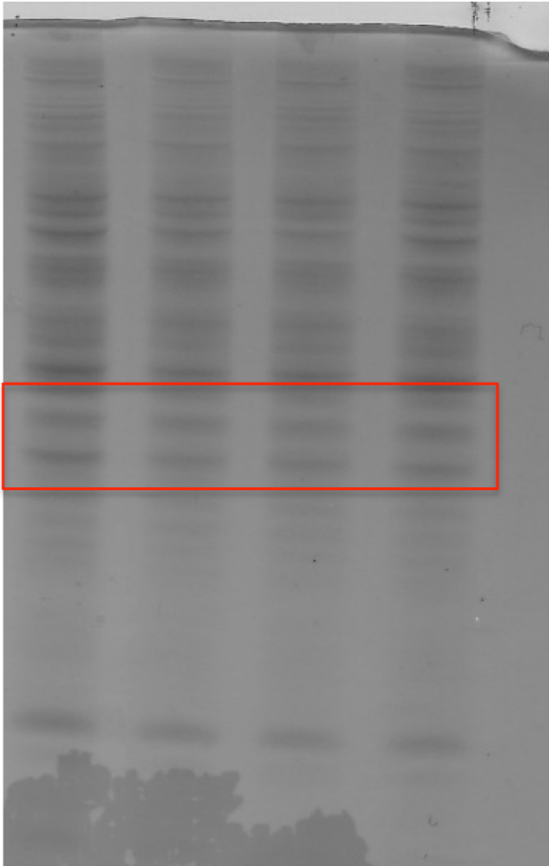

COOMASSIE  
STAINED GELS

RAW DATA Figure 5C

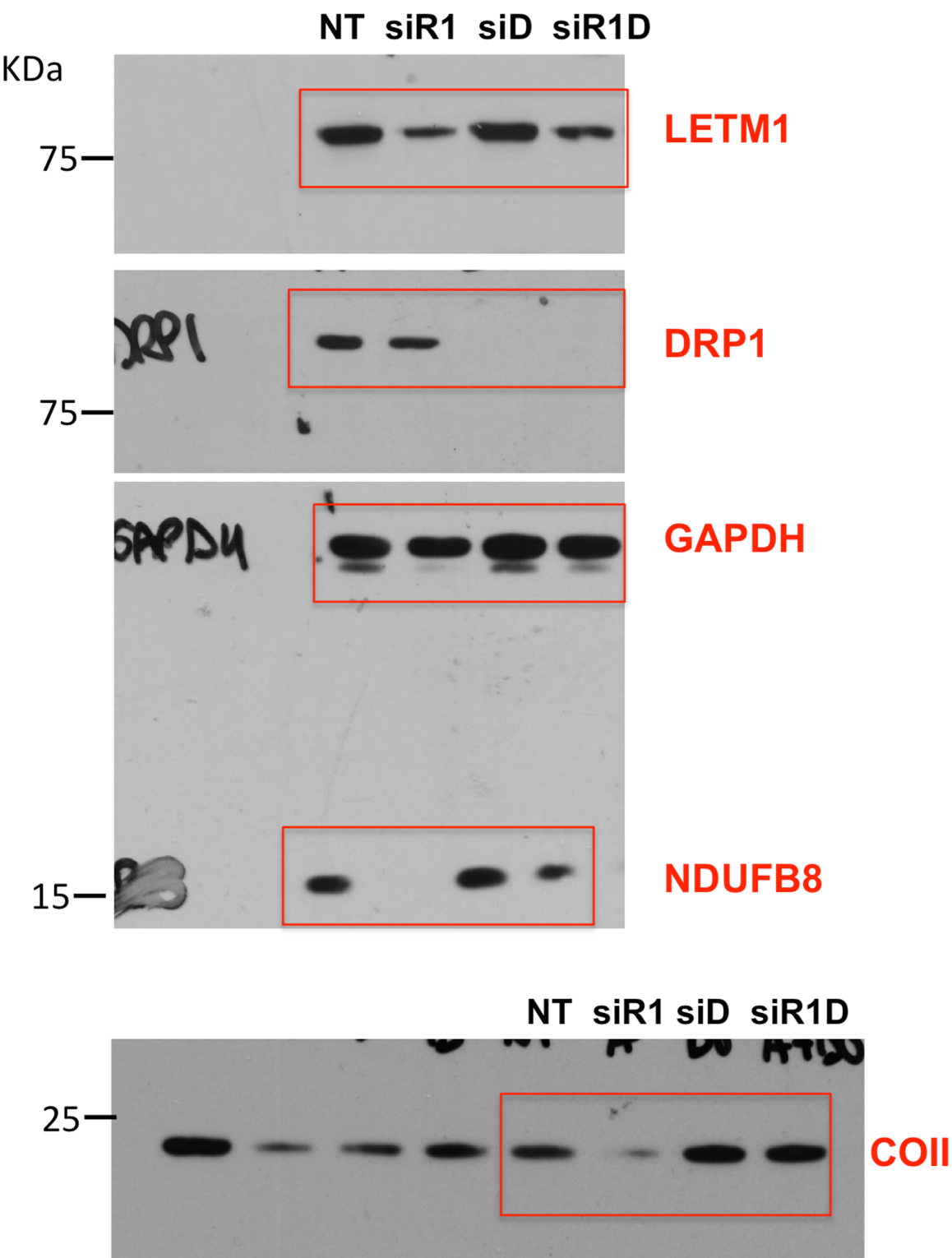

Supplement: Supplementary file 11 — Source Data for Figure 5 [file EMMM-10-e8550-s010.pdf]

# RAW DATA Figure 6A

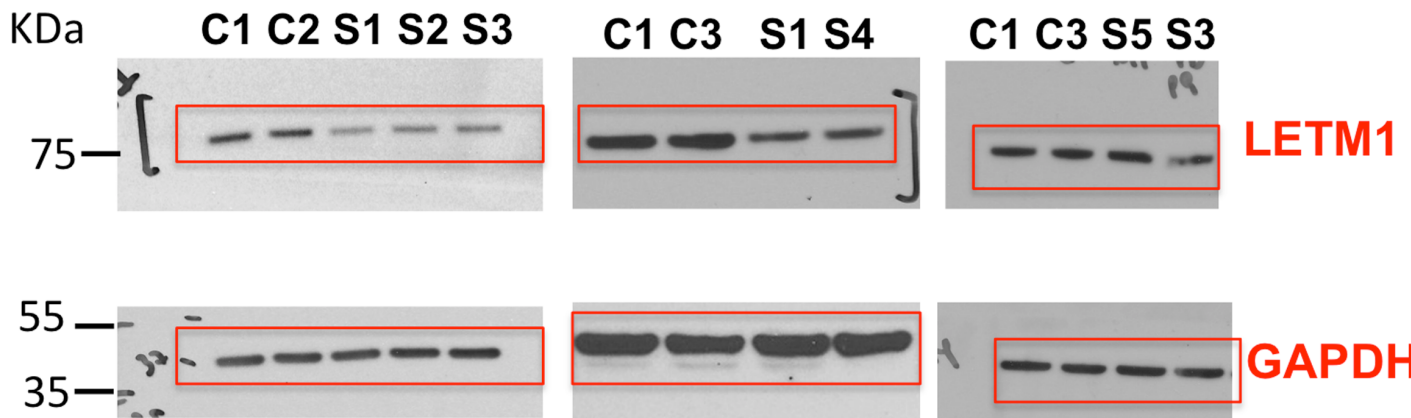

Supplement: Supplementary file 12 — Source Data for Figure 6 [file EMMM-10-e8550-s011.pdf]

RAW DATA Figure 7E

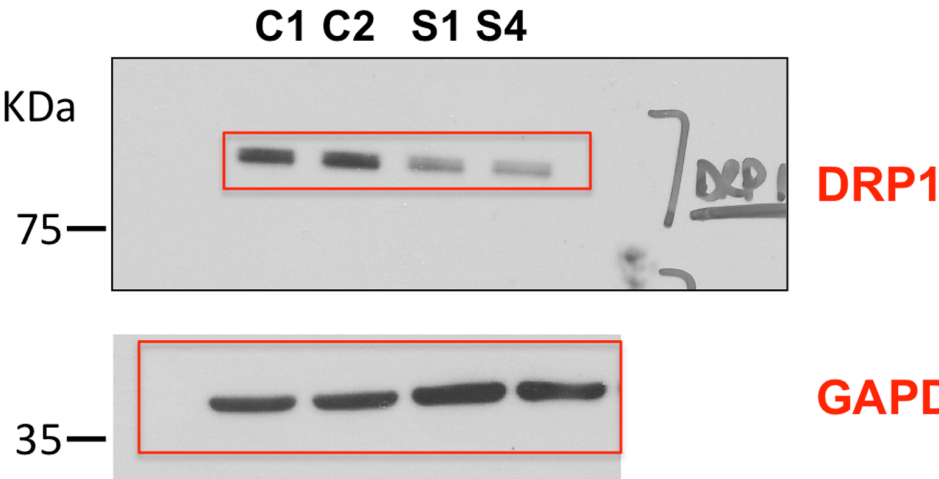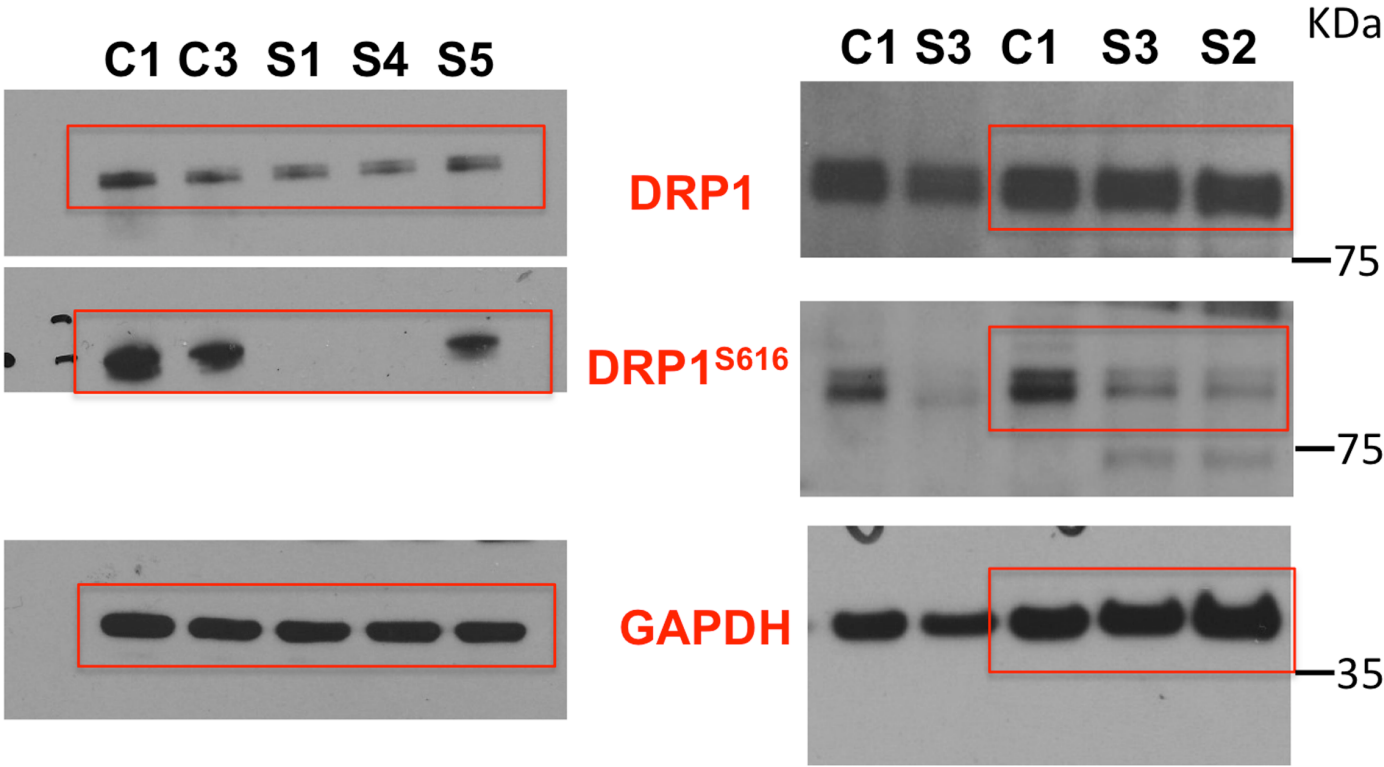

Supplement: Supplementary file 13 — Source Data for Figure 7 [file EMMM-10-e8550-s012.pdf]

# RAW DATA Figure 8A

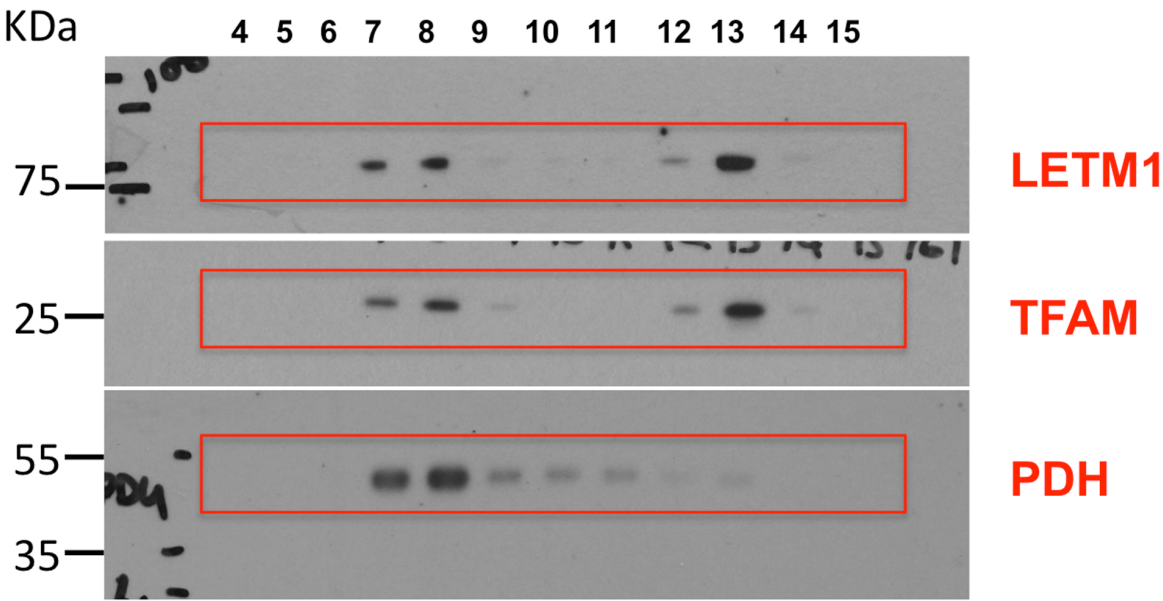

RAW DATA Figure 8B

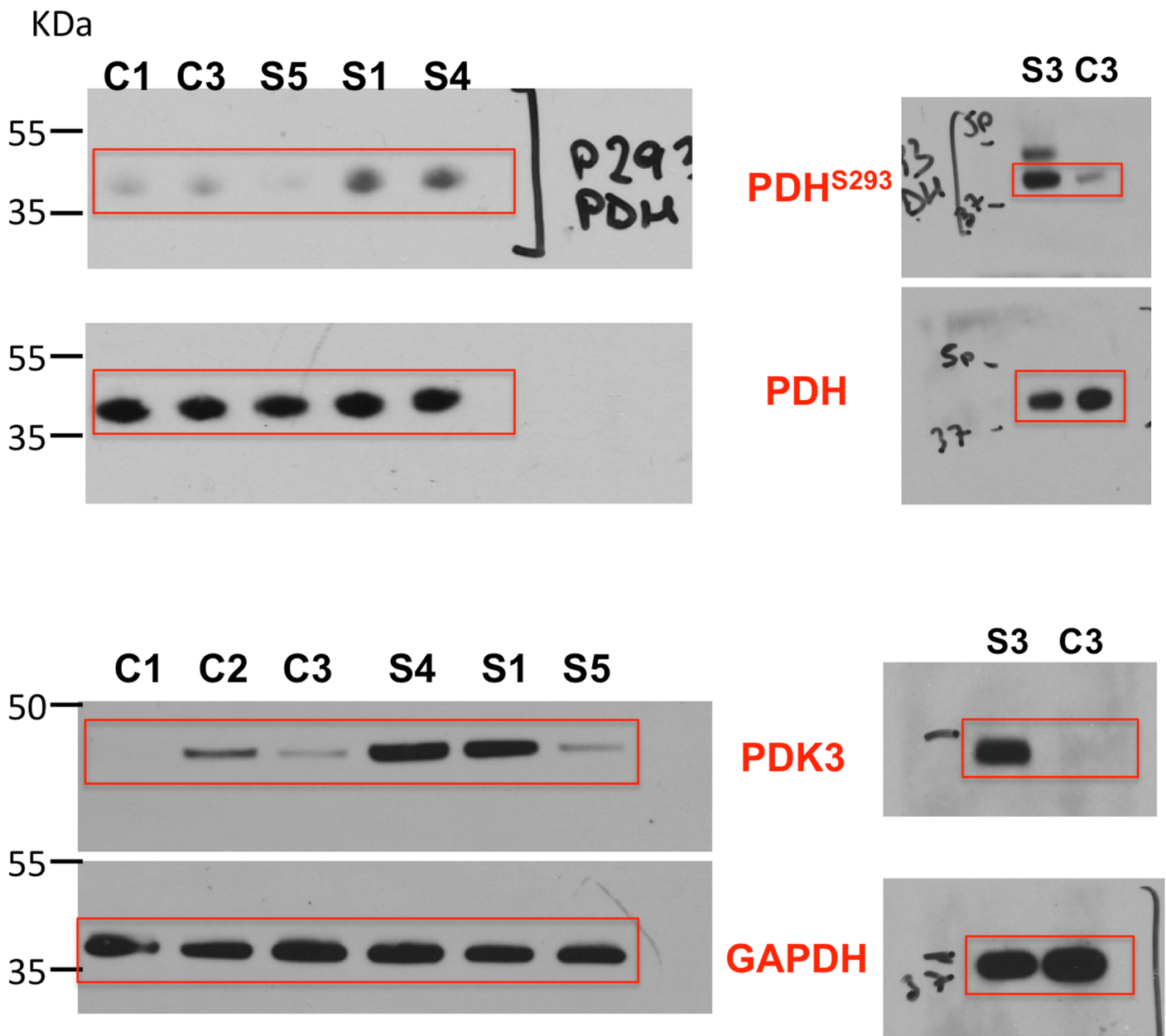

Supplement: Supplementary file 14 — Source Data for Figure 8 [file EMMM-10-e8550-s013.pdf]

# RAW DATA Figure 9B

HG AA BHB HG AA BHB

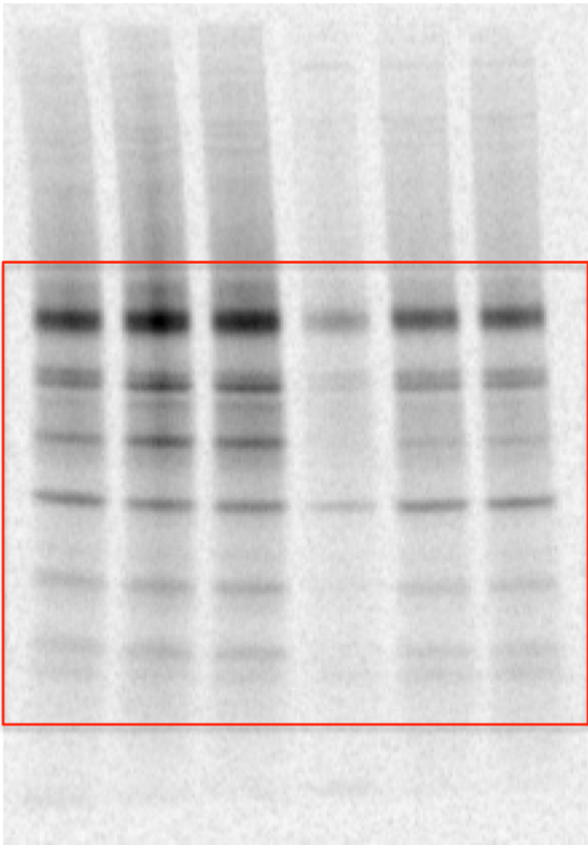

**<sup>35</sup>S labeling**

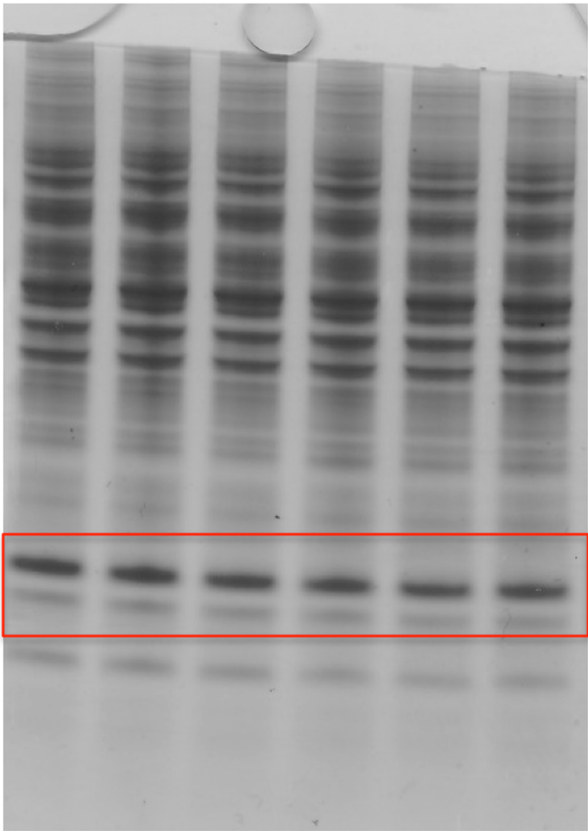

**COOMASSIE  
STAINED GEL**

75—

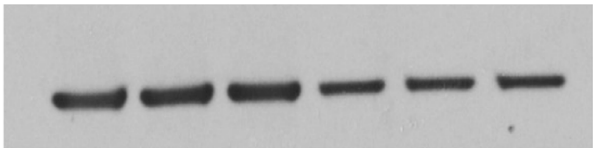

**LETM1**

Supplement: Supplementary file 15 — Source Data for Figure 9 [file EMMM-10-e8550-s014.pdf]
