# Supplementary material for: LETM1 couples mitochondrial DNA metabolism and nutrient preference
Source: EMBO Mol Med. 2018 Jul 16;10(9):e8550. doi: 10.15252/emmm.201708550 (PMC6127893; doi:10.15252/emmm.201708550)

RAW DATA Appendix Figure 1B

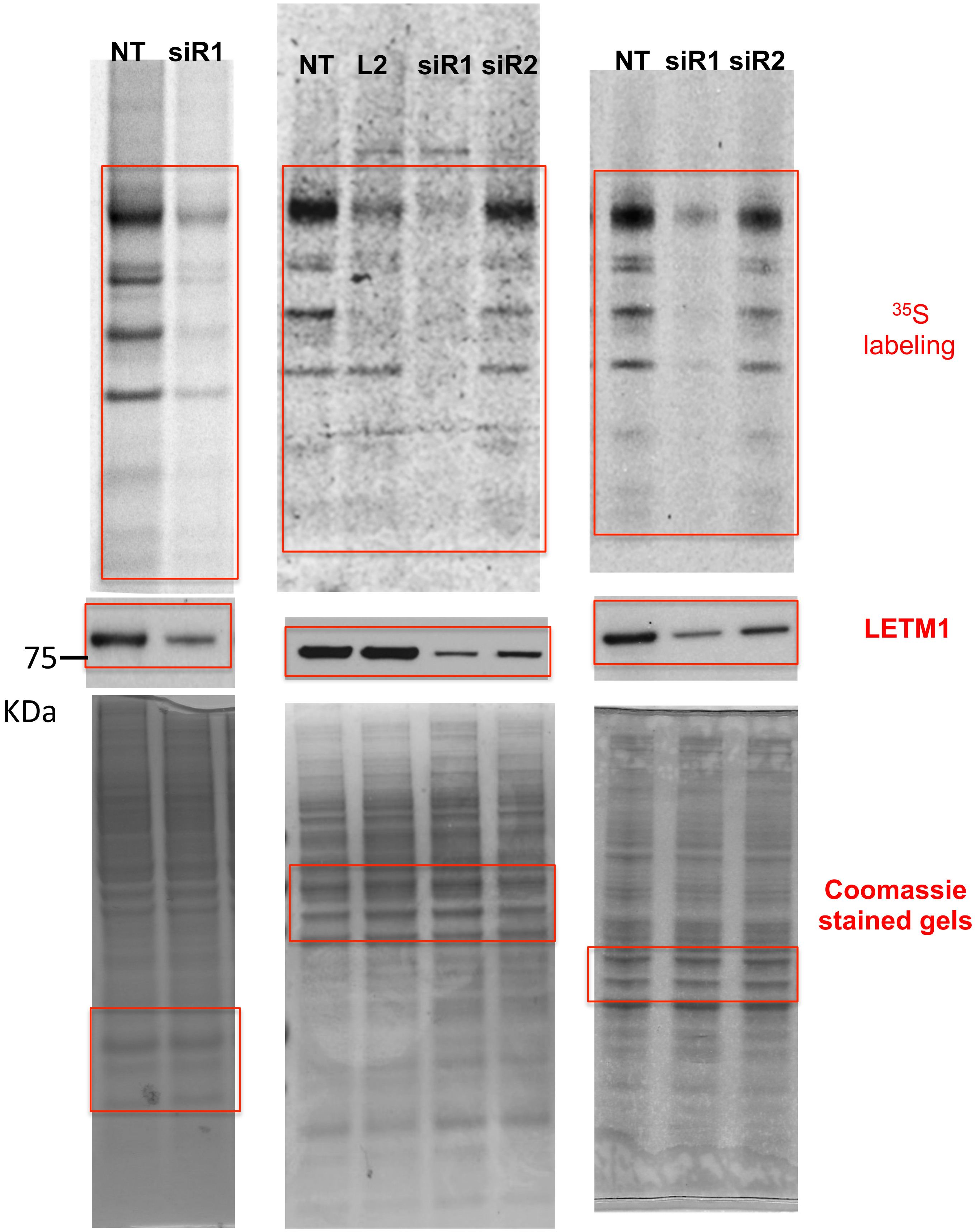

RAW DATA Appendix Figure 1 C

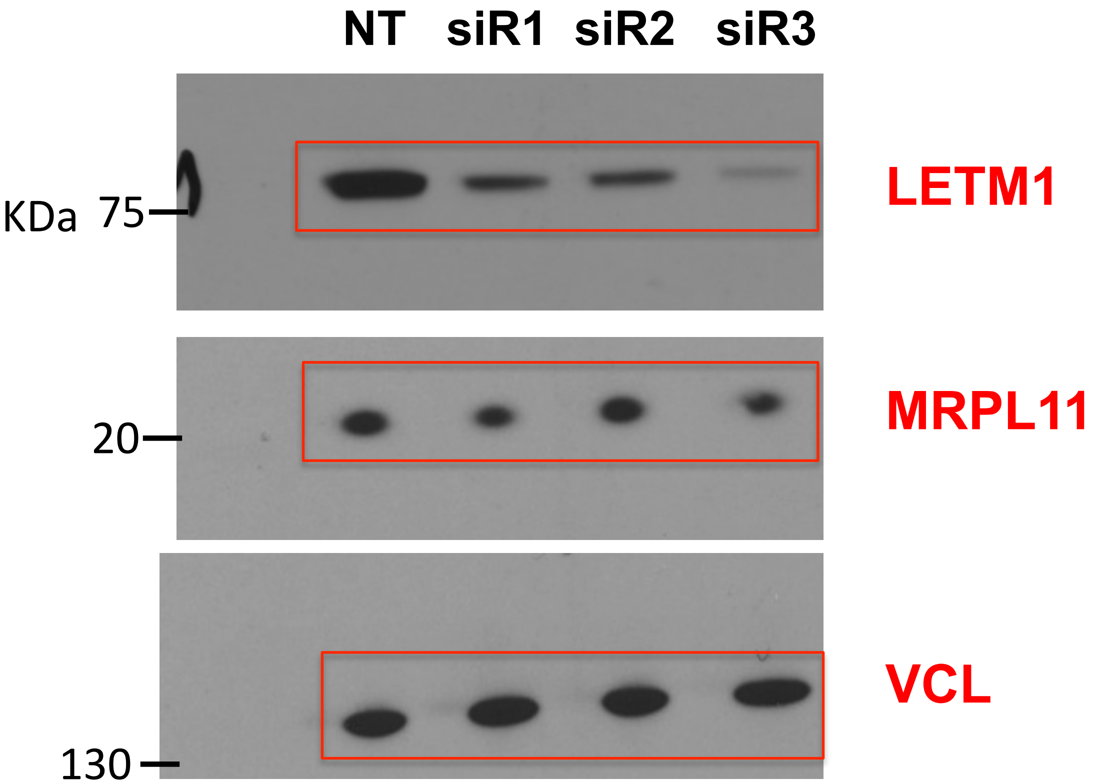

RAW DATA Appendix Figure 3 A

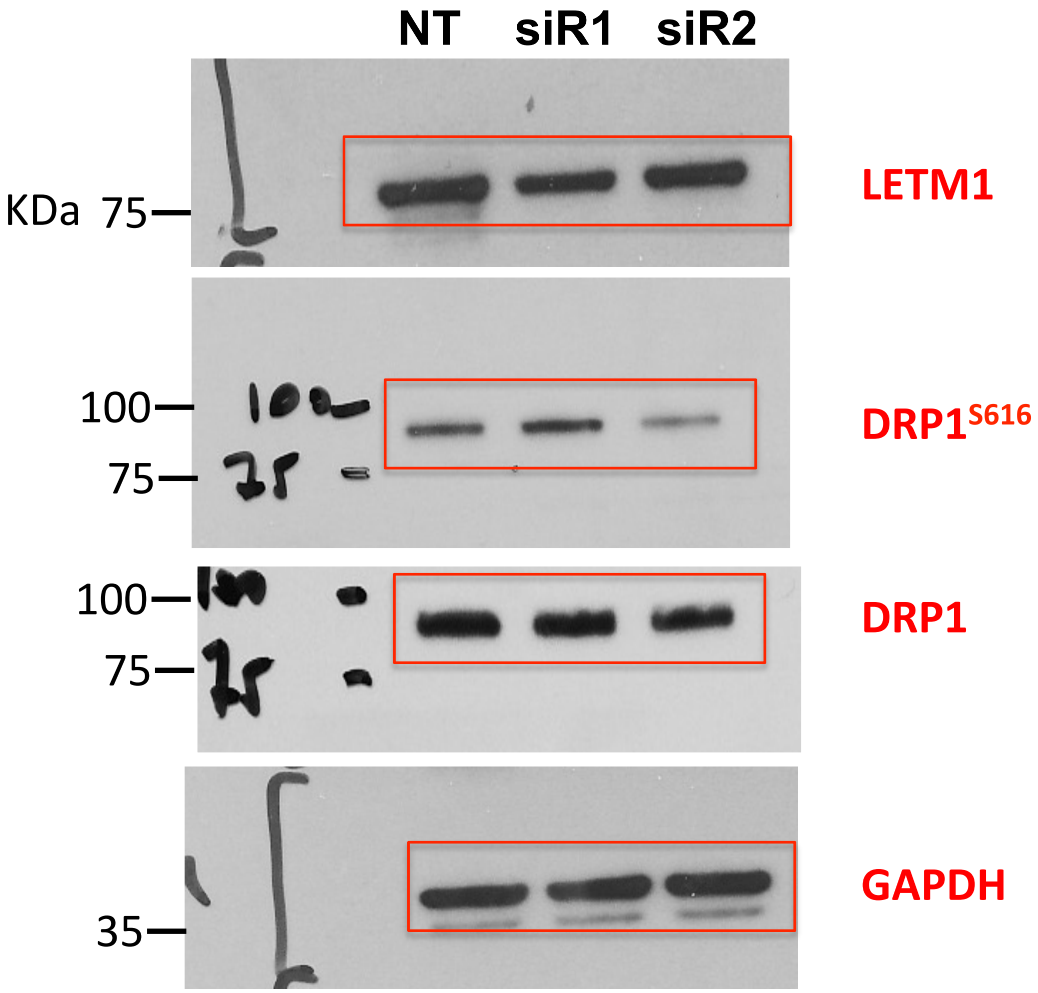

RAW DATA Appendix Figure 3 B

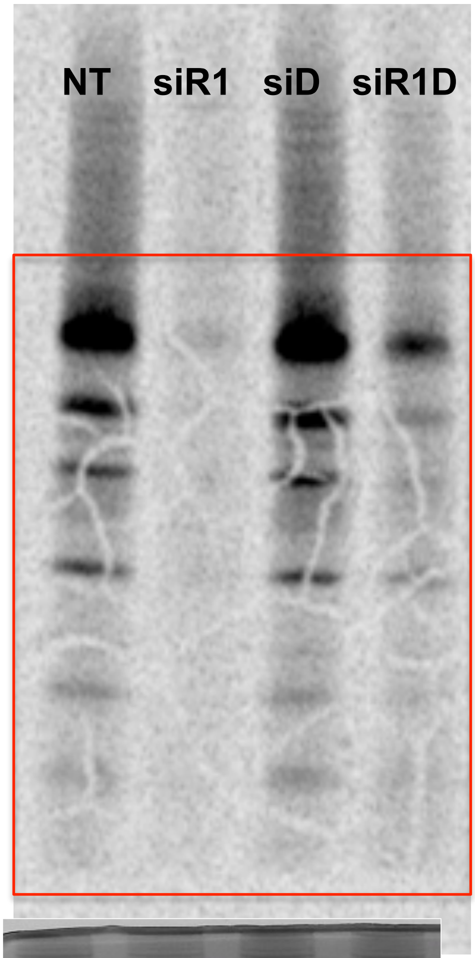

^{35}S labeling

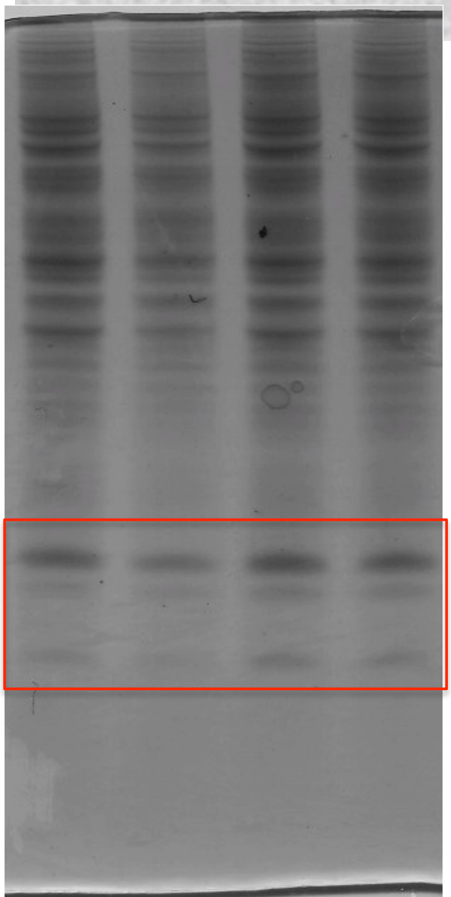

Coomassie stained gels

RAW DATA Appendix Figure 5 A

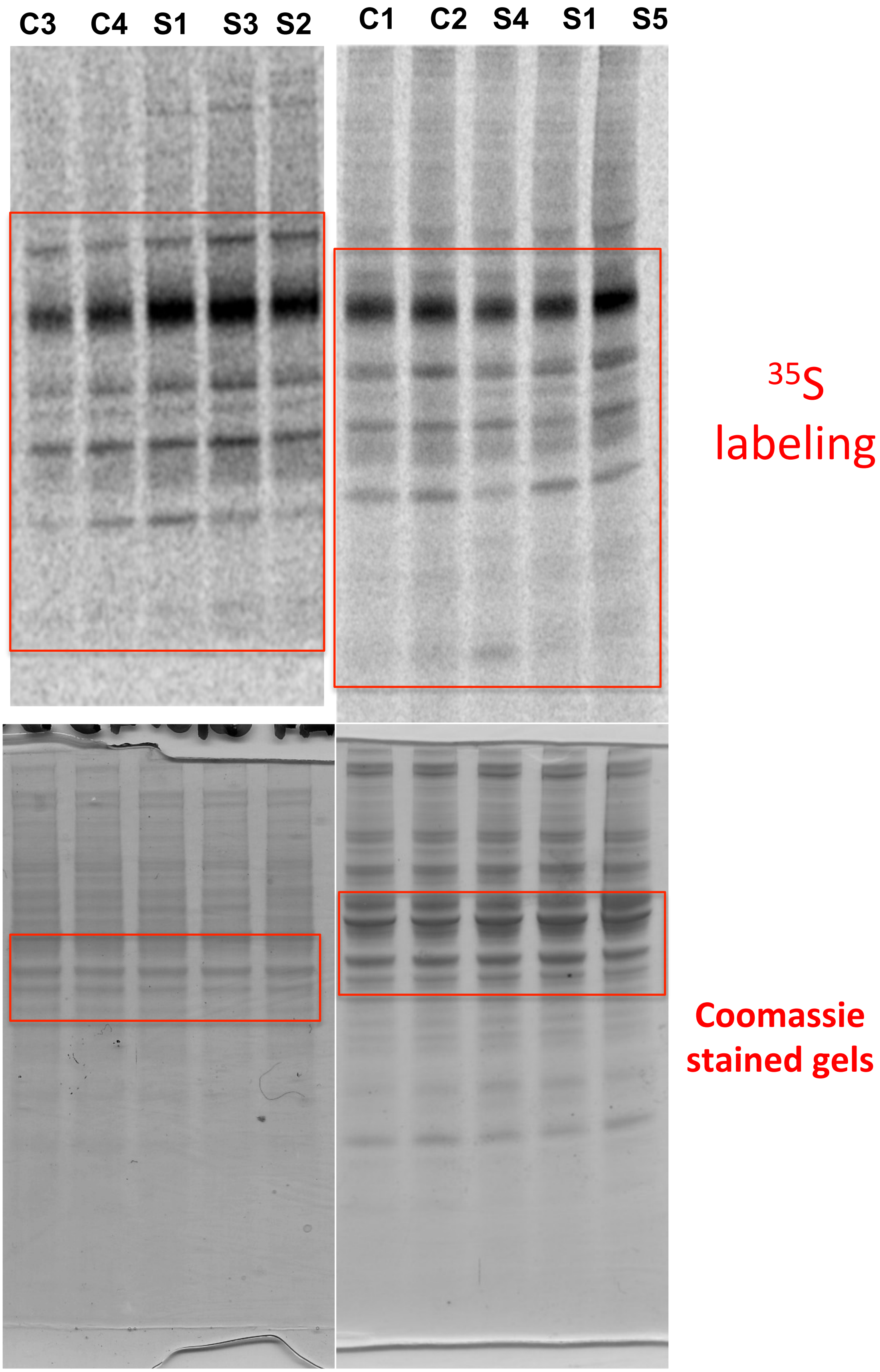

RAW DATA Appendix Figure 5 B

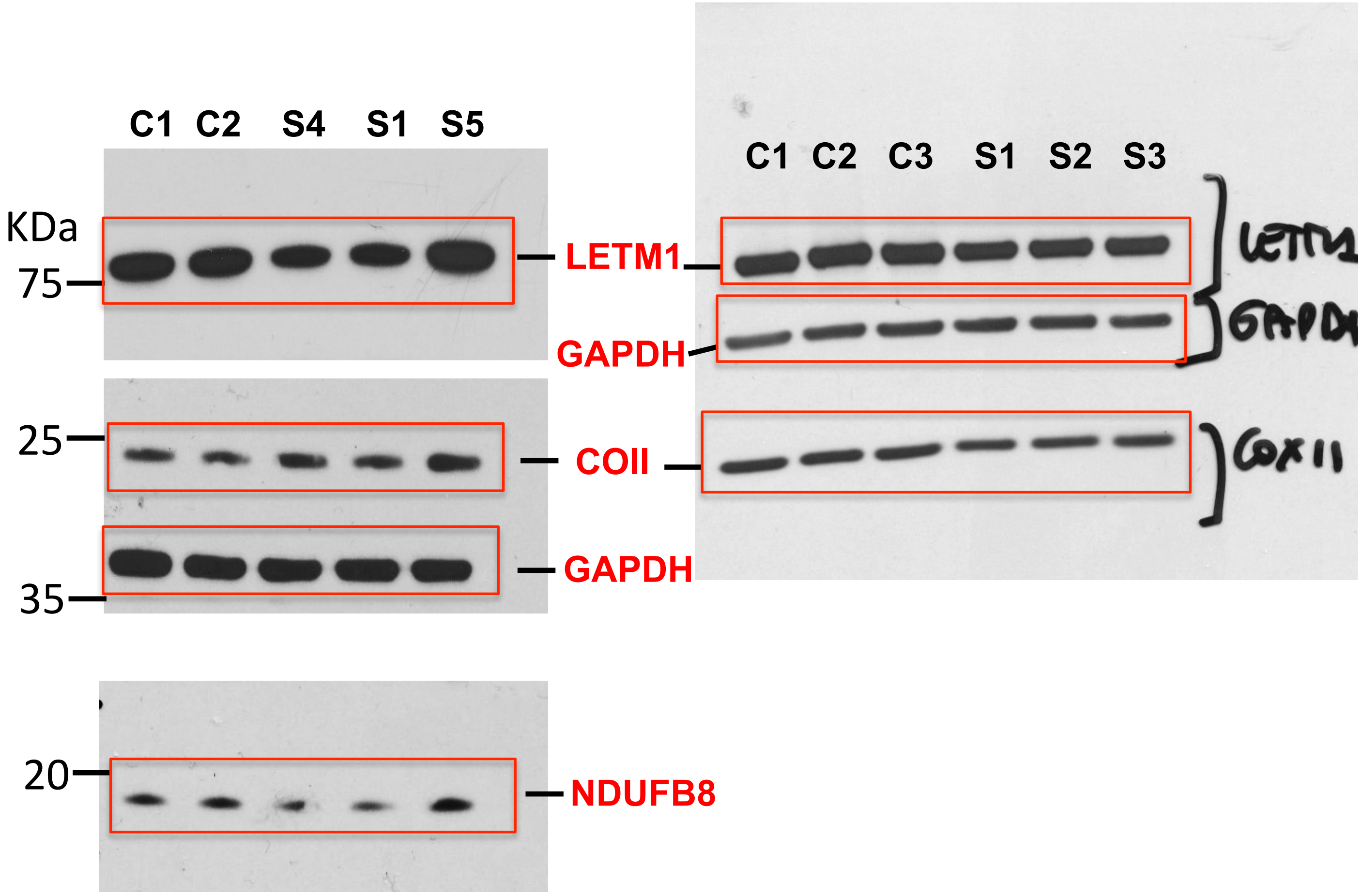

RAW DATA Appendix Figure 7

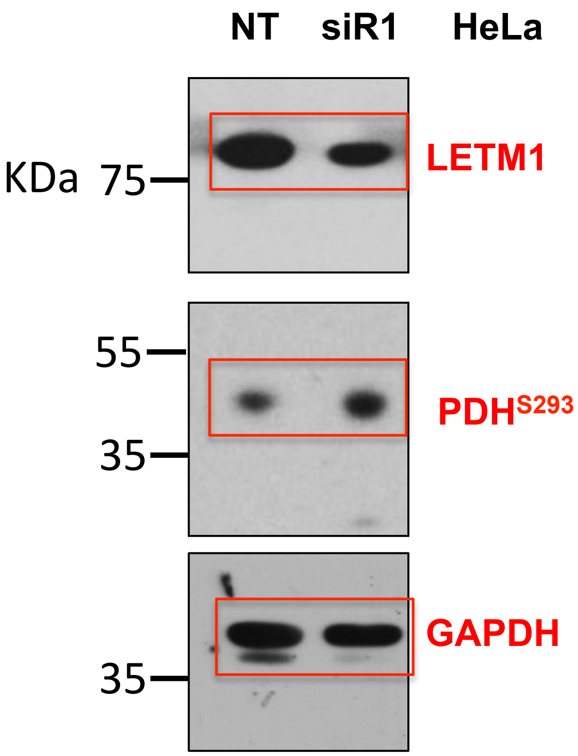

Supplement: Supplementary file 9 — Source Data for Figure 3 [file EMMM-10-e8550-s008.pdf]
